# Trehalose–Carnosine Prevents the Effects of Spinal Cord Injury Through Regulating Acute Inflammation and Zinc(II) Ion Homeostasis

**DOI:** 10.1007/s10571-022-01273-w

**Published:** 2022-09-19

**Authors:** Irene Paterniti, Alessia Filippone, Irina Naletova, Valentina Greco, Sebastiano Sciuto, Emanuela Esposito, Salvatore Cuzzocrea, Enrico Rizzarelli

**Affiliations:** 1 Department of Chemical, Biological, Pharmaceutical and Environmental Science, University of Messina, Viale Ferdinando Stagno D’Alcontres, 31-98166 Messina, Italy; 2 Institute of Crystallography, National Council of Research, CNR, Via Paolo Gaifami 18, 95126 Catania, Italy; 3 National University Consortium Metals Chemistry in Biological Systems (CIRCMSB), Via Celso Ulpiani, 27-70126 Bari, Italy; 4 Department of Chemical Sciences, University of Catania, Viale A. Doria 6, 95125 Catania, Italy

**Keywords:** Spinal cord injury, Inflammation, Apoptosis, Neurotrophic factors, Ion homeostasis

## Abstract

Spinal cord injury (SCI) leads to long-term and permanent motor dysfunctions, and nervous system abnormalities. Injury to the spinal cord triggers a signaling cascade that results in activation of the inflammatory cascade, apoptosis, and Zn(II) ion homeostasis. Trehalose (Tre), a nonreducing disaccharide, and l-carnosine (Car), (β-alanyl-l-histidine), one of the endogenous histidine dipeptides have been recognized to suppress early inflammatory effects, oxidative stress and to possess neuroprotective effects. We report on the effects of the conjugation of Tre with Car (Tre–car) in reducing inflammation in in vitro and in vivo models. The in vitro study was performed using rat pheochromocytoma cells (PC12 cell line). After 24 h, Tre–car, Car, Tre, and Tre + Car mixture treatments, cells were collected and used to investigate Zn^2+^ homeostasis. The in vivo model of SCI was induced by extradural compression of the spinal cord at the T6–T8 levels. After treatments with Tre, Car and Tre–Car conjugate 1 and 6 h after SCI, spinal cord tissue was collected for analysis. In vitro results demonstrated the ionophore effect and chelating features of l-carnosine and its conjugate. In vivo, the Tre–car conjugate treatment counteracted the activation of the early inflammatory cascade, oxidative stress and apoptosis after SCI. The Tre–car conjugate stimulated neurotrophic factors release, and influenced Zn^**2+**^ homeostasis. We demonstrated that Tre–car, Tre and Car treatments improved tissue recovery after SCI. Tre–car decreased proinflammatory, oxidative stress mediators release, upregulated neurotrophic factors and restored Zn^2+^ homeostasis, suggesting that Tre–car may represent a promising therapeutic agent for counteracting the consequences of SCI.

**Supplementary Information:**

The online version contains supplementary material available at 10.1007/s10571-022-01273-w.

## Introduction

**Scheme 1:**
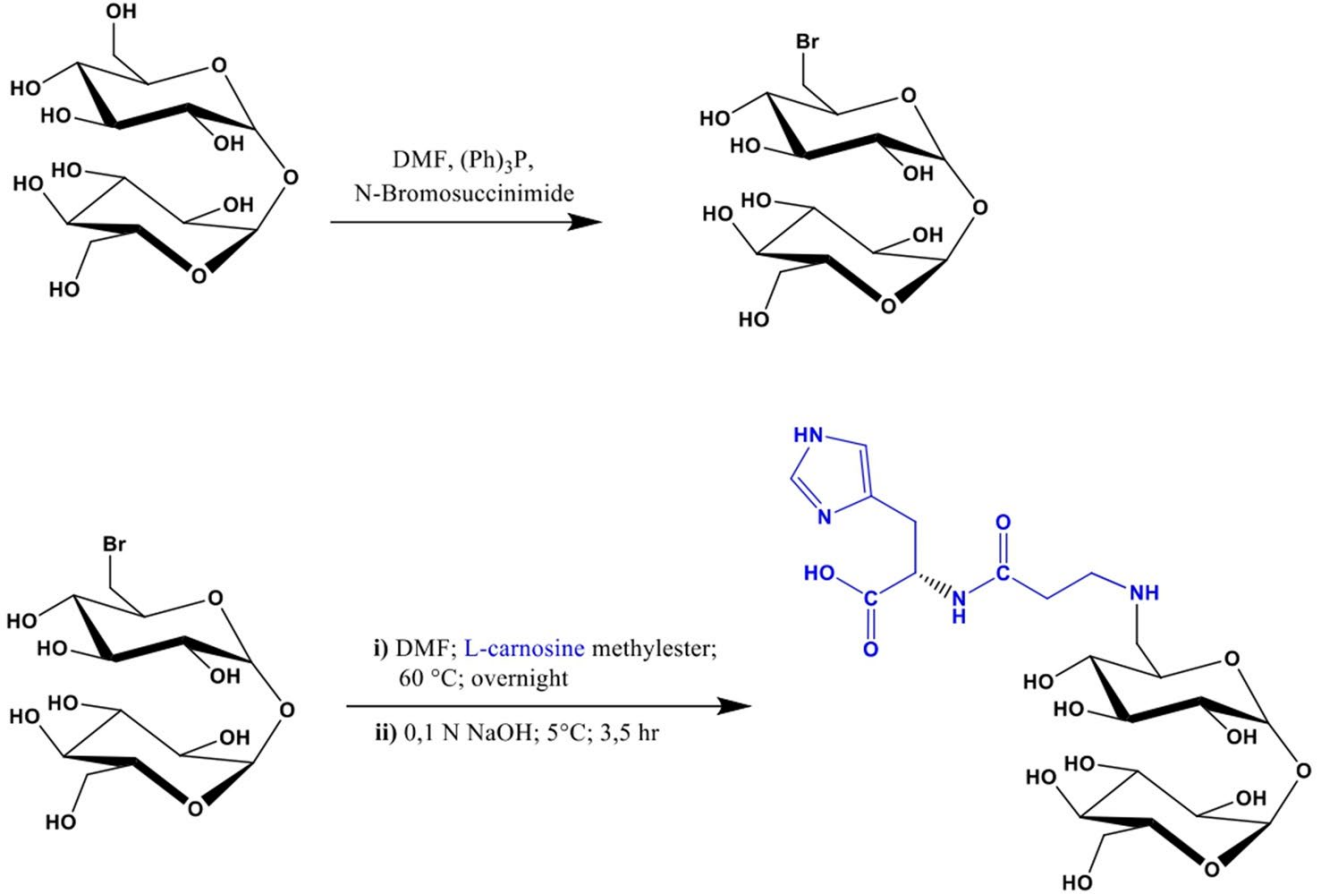
Synthesis of trehalose–carnosine

Spinal cord injuries (SCIs) are severe, life-threatening medical conditions that alter the physical and psychological conditions of patients (Vural et al. 2020). SCI exhibits a global incidence of 10.5 cases per 100,000 people with consequent high costs (Kumar et al. 2018; Cao et al. 2011). Severe mechanical injury to the spinal cord mimics the pathophysiology of SCI; it causes tissue damage (Stahel et al. 2012), blood–brain barrier disruption, haemorrhage, oedema, axonal destruction and cell membrane alterations (Kwiecien 2021). The second step of injury, referred to as secondary SCI, involves activation of a number of cellular and molecular processes concerning (1) the formation of free radicals (Hall and Braughler 1993), (2) oxidative and nitrosative stress (Bains and Hall 2012) (3) delayed calcium influx (Du et al. 1999), (4) immune system response and (5) increased cytokines, with the upregulation of inflammatory, autophagic and apoptotic agents (Abbaszadeh et al. 2020; Aidemise Oyinbo 2011; Ludwig et al. 2017). Reactive oxygen species (ROS) and reactive nitrogen species (RNS) together with inflammatory mediators tune matrix metalloproteinases (MMPs), a large family of zinc-bound extracellular proteases that facilitate glial scar formation in the injured spinal cord (Hsu et al. 2008). Specifically, MMP-2 and MMP-9 are involved in secondary SCI through degradation of basal components of the blood spinal cord barrier (Noble et al. 2002). SCI induces extensive nerve cell apoptosis and necrosis following the secondary injury that disrupts the microenvironment of axon regeneration (Tran et al. 2018); apoptosis is also considered the primary process responsible for partial or complete loss of motor and sensory functions (Beattie et al. 2000; Ray 2020). After the initial mechanical injury and secondary SCI, long-standing progressive neurodegeneration occurs, and neurons fail to transmit electrical and chemical signals losing their outgrowth capacity (Aidemise Oyinbo 2011). SCI lacks effective therapeutics and exhibits poor healing. Currently, the main treatment for SCI is surgery combined with treatment with methylprednisolone sodium succinate (MP), which remains the most commonly administered drug after acute SCI (Evaniew et al. 2015; Fehlings et al. 2017). Although MP is an anti-inflammatory agent that can inhibit lipid peroxidation (Bracken 2001), it can cause serious trauma and many side effects and does not ameliorate neurite sprouting, remyelination of axons or hence, functional recovery (Ito et al. 2009). Therefore, the development of novel pharmacological agents for the successful and safe treatment of SCI is a priority for clinical practice. β-alanyl-l-histidine, a natural dipeptide known as carnosine (Car) (Gulewitsch and Amiradžibi 1900) is primarily found in skeletal muscle, but it is also present at mM concentrations in the olfactory bulb of mammals (Boldyrev et al. 2013). This endogenous dipeptide is a pH buffering agent (Posa and Baba 2020) and protects cells from ROS, RNS and reactive carbonyl species (RCS) damage by means of the histidine imidazole ring and the amino terminus of the β-alanine residue (Pavlov et al. 1993; Aldini et al. 2005; Nicoletti et al. 2007). l-carnosine forms different complex species with metal ions [copper(II) and zinc(II) ions] (Dobbie and Kermack 1955) and its chelating ability induces different protective functions (Trombley et al. 2000; Kawahara et al. 2020). Among its different pleiotropic abilities (Cuzzocrea et al. 2007; Oppermann et al. 2019; Zhao et al. 2019; Corona et al. 2011; Spina-Purrello et al. 2010; Miceli et al. 2018; Caruso et al. 2019; Jain et al. 2020; Boakye et al. 2019; Attanasio et al. 2013, 2009; Bellia et al. 2011; Greco et al. 2020), this dipeptide displays neuroprotective features, as attested by the reduced brain damage and improved functional outcomes observed in mouse models of focal ischaemic stroke (Rajanikant et al. 2007; Baek et al. 2014). Furthermore, l-carnosine is a good candidate for a successful and reliable agent for SCI models in rodents (Di Paola et al. 2011; Albayrak et al. 2015) due to its protective effects against inflammation (Kubota et al. 2020), brain oxidative stress, apoptosis and autophagy (Xie et al. 2017). However, the potential therapeutic action of Car is drastically hampered by its hydrolysis due to serum (Teufel et al. 2003; Bellia et al. 2014) and tissue (Lenney et al. 1985) carnosinase enzymes. Serum degradation of the dipeptide can be prevented by the use of (1) carriers (Kim et al. 2020), (2) d-carnosine (Di Paola et al. 2011), or (3) l-carnosine derivatives (Bellia et al. 2012; Menini et al. 2019). Car derivatives and their conjugates with different polysaccharides can block or delay dipeptide degradation (Bellia et al. 2012). Different reports on the behavior of Car conjugates with trehalose (Tre) show that this disaccharide not only protects l-carnosine from hydrolysis induced by carnosinase (Rizzarelli et al. 2007) but also potentiates the protective functions of the dipeptide (Grasso et al. 2017) and retains the metal ion complex species formation of the dipeptide (Grasso et al. 2011). Trehalose (see Scheme 1) (1-α-d-glucopyranosyl-α-d-glucopyranoside) is a stable, soluble and nonreducing disaccharide detected in many lower-order organisms (Elbein et al. 2003), including yeast, fungi, invertebrates and plants, but is not present in mammals (Tapia and Koshland 2014; Wiemken 1990). In addition, the disaccharide does not exert toxicity despite the high concentrations usually tested (Richards et al. 2002). It is a preserving and stabilizing agent for cell membranes under stress conditions, such as high temperature, freezing, osmotic shock, and dehydration (Crowe et al. 1983) and an effective molecule in preventing protein aggregation (Liu et al. 2005; Attanasio et al. 2007). Furthermore, recent reports have shown that trehalose not only inhibits inflammatory and oxidative stress (Minutoli et al. 2008; Echigo et al. 2012) but also acts as an autophagy enhancer and chemical chaperone as indicated in different in vitro and in vivo assays (Fewell et al. 2014; Casarejos et al. 2011), and it increases brain zinc levels in a mouse model of traumatic brain injury (Portbury et al. 2018). Although the exact biochemical pathways involved in trehalose’s effects on mammalian cells, including neuronal cells, are not yet understood, the ability of disaccharides to protect against SCI has been previously reported (Iturriaga et al. 2009; Martano et al. 2017). In addition to antioxidant and anti-inflammatory activities (Nazari-Robati et al. 2019), Tre induces MMP expression and the activation of some heat shock proteins and neurotrophins (NTs), such as BDNF (Nazari-Robati et al. 2019; Nasouti et al. 2019; Liang et al. 2018). Overall, the sharing of many potential protective properties by Car and Tre against SCI prompted us to examine the ability of the conjugate trehalose–carnosine (Tre–car) to act as an antioxidant, anti-inflammatory and anti-apoptotic agent in an SCI mouse model. Furthermore, the effects of the metal binding ligand and ionophore molecule features of Tre–car and of its crosstalk with Zn^2+^ were investigated. Zinc is abundant in the spinal cord, where it participates in several physiological and pathophysiological processes, including neurotransmission, SCI, and amyotrophic lateral sclerosis. However, the mechanisms underlying zinc homeostasis in the spinal cord remain largely unknown (Zong et al. 2017). Zn^2+^ is able to act as an intracellular regulator of major signalling pathways, and its dyshomeostasis induces aberrant expression of different factors in multiple pathologies (Milardi and Rizzarelli 2011). Zinc ion signaling occurs through at least twenty-four membrane transporters (14 Zrt, Irt-like proteins (ZIP) zinc importers and 10 zinc transporters (ZnT) zinc exporters), metallothioneins (MTs), and a zinc-sensing transcription factor, metal-response element (MRE)-binding transcription factor-1 (MTF-1) (Kambe et al. 2015). Among the ZnT family members, ZnT1 is the most ubiquitously expressed, being responsible for the efflux transporter of zinc, and is the only member found on the plasma membrane (Kambe et al. 2015). SCI modifies ZnT-1 mRNA levels, which are related to BDNF mRNA levels (Wang et al. 2011b; Qin et al. 2006). Zn^2+^ supplementation reduces neuronal apoptosis after SCI, and the acute phase serum zinc concentration is a reliable biomarker for predicting functional outcomes after SCI (Li et al. 2019a; Kijima et al. 2019). Different reports highlight the protective role of this metal ion on SCI (Wen et al. 2021).

## Materials and Methods

Anhydrous α,α-trehalose and silica gel 60 F254 plates were purchased from Merck Co. l-carnosine, N-bromosuccinimide, triphenylphosphine, acetyl chloride, anhydrous dimethylformamide and anhydrous methanol were purchased from Sigma Aldrich Co.

All compounds used for in vitro and in vivo studies were purchased from Sigma–Aldrich Co. (Poole, United Kingdom). All solutions used for in vivo infusions were prepared using nonpyrogenic saline (0.9% wt/vol NaCl; Baxter Health care Ltd., Thetford, United Kingdom). All antibodies used for western blot analysis, immunohistochemistry (IHC) and immunofluorescence (IF) were purchased from Santa Cruz Biotechnology (Texas, USA), Cell Signalling Technology (Massachusetts, USA) and Abcam (Cambridge, UK).

### Synthesis and Characterization of Trehalose–Carnosine Conjugate

The synthesis of trehalose–carnosine (Tre–car, Scheme 1) was performed following synthetic routes previously reported in the literature and appropriately adapting them to better match our preparative needs at scale. Briefly, a mixture of 6-bromo-6-deoxy-α,α-trehalose and 6,6′-dibromo-6,6′-dideoxy-α,α-trehalose was obtained by treating a solution of trehalose and triphenylphosphine in DMF with N-bromosuccinimide (Hanessian and Lavallee 1972), from which, after removing the excess reagents, 6-bromo-6-deoxy-α,α-trehalose was isolated by PLC on an RP-8 column (Grasso et al. 2013). This bromine derivative, dissolved in DMF, was then left to react overnight at 60 °C with l-carnosine methyl ester, prepared as previously reported (Rizzarelli et al. 2007). Following this, the methyl ester group of the l-carnosine moiety was hydrolyzed by treating the reaction mixture with sodium hydroxide of 0.1 N final concentration for 3.5 h at 5 °C. After neutralization with HCl, the solvents were removed under vacuum, and the residue, taken up with water, was loaded onto a column of Dowex^®^-50 resin (H^+^ form). The column was then eluted with a gradient of HCl from 0 to 0.25 N, and the progress of the fractionation was monitored by TLC [SiO_2_, 2-propanol/ammonia solution (32%), 70:30 v/v; Tre–car Rf = 0.2] using Fast red salt B as a chromogenic reagent. Fractions containing chromatographically pure Tre–car were pooled, repeatedly reduced to a small volume and then lyophilized over potassium hydroxide {14% overall yield; ESI/MS (direct injection of aqueous methanol solution), m/z 551.2 [M + H]^+^}. Before use in biological experiments, the conjugate was subjected to ^1^H-NMR spectroscopic analysis to ensure its degree of purity.

^1^H-NMR spectra were recorded using a Varian Unity Inova spectrometer at 500 MHz. The experiments were performed in D_2_O at 27 °C, and the chemical shifts are reported as δ (ppm) related to the resonance of residual HOD. VnmrJ v2.0 software was used to process the data. ESI–MS spectra were obtained using an Agilent Technologies 6410 Triple Quad LC/MS equipped with a Multimode (ESI/APCI) source.

### In Vitro Experiments

#### Cell Line

Rat pheochromocytoma (PC12) cells were obtained from the American Type Culture Collection (ATTC, Manassas, VA) and cultured at 12 passages in PRMI1640 medium supplemented with 10% horse serum (HS), 5% foetal bovine serum (FBS), 2 mM L-glutamine and 1% (v/v) penicillin (100 units/ml)/streptomycin (100 mg/ml). This medium contains zinc at submicromolar concentration, as determined by ICP-MS (data not shown). Cells were cultured in a humidified atmosphere of air/CO_2_ (95:5) at 37 °C in an incubator (Heraeus Hera Cell 150).

#### Cellular Staining and Fluorescent Microscopy Imaging

The intracellular levels of labile zinc cations were measured as the fluorescence emission of cells upon loading them with the membrane-permeant zinc specific indicator 2-[2-[2-[2-[bis(carboxylatomethyl)amino]-5-methoxyphenoxy]ethoxy]-4-(2,7-difluoro-3-oxido-6-oxo-4a,9a-dihydroxanthen-9-yl)anilino]acetoxymethyl, FluoZin™-3 (ThermoFisher Scientific), using fluorescence microscopy. For fluorescent microscopy imaging studies, PC12 cells were seeded on l-poly-lysinated glass bottom dishes with 22 mm of glass diameter (WillCo Wells B.V., Amsterdam—NL) at a density of 25 × 10^4^ per dish in RPMI1640 complete medium until cellular adhesion was obtained. Thereafter, cells were treated with 5 mM Car, Tre, Tre + Car mixture, Tre–car, 20 μM or 50 μM Zn(II) or 50 μM dipicolinic acid (DPA), a membrane-impermeable zinc chelator, in complete RPMI1640 medium supplemented with 1% HS and 0.5% FBS. After 20 h of treatment cells were rinsed with serumfree medium and stained by 1 h incubation with FluoZin™-3, acetoxymethyl (AM) cell permeant zinc indicator (ThermoFisher Scientific) at a final concentration of 1 μM from 1 mM stock solution in DMSO and the cell-permeant nuclear counterstain Hoechst33342 (NucBlue^®^ Live ReadyProbes^®^ Reagent, Life Technologies), followed by buffer rinsing (2 × 1 ml). As a baseline to exclude cell auto-fluorescence, PC12 cells were treated only with Hoechst33342 without FluoZin-3. After staining, cells were fixed in fresh 4% paraformaldehyde and deeply rinsed (3 × 2 ml) with PBS. Images were analysed under a Leica DMI 6000B epifluorescence inverted microscope with Adaptive Focus Control with 63 × magnification. Images were taken at random locations throughout the area of the well for all of the samples. Images analysis was carried out by using LAS X Life Science Microscope Software and the fluorescence emission was normalized to the number of cells presented in each field.

#### Western Blot Analysis

Cells were treated for 24 h with 5 mM carnosine, trehalose, trehalose + carnosine mixture or trehalose–carnosine in complete RPMI1640 medium. Therefore, cells were harvested with RIPA buffer containing Halt Protease and Phosphatase Inhibitor Single-Use Cocktail (ThermoFisher), lysates were separated by SDS–PAGE on 4–15% precast gels, transferred to nitrocellulose membranes and treated with blocking buffer at room temperature for 1 h followed by incubation with primary antibodies overnight at 4 °C. Anti-ZnT1 antibody (Cat# ARP44019, 1:1000 dilution) was purchased from Aviva Systems Biology (CA, USA). Anti-GAPDH (Cat# ab8245, 1:2000 dilution) was purchased from Abcam (MA, USA). Next, membranes were incubated for 1 h with goat anti-rabbit (Cat# 926-68071) or anti-mouse (Cat# 926-68070) antibodies labeled with IRDye 680 (1:20,000 dilution, LI-COR Biosciences) and used for IRS1 immunoblots, and hybridization signals were detected using the Odyssey Infrared Imaging System (LI-COR Biosciences). Western blot data were quantified using densitometric analysis of the hybridization signals in three different blots per experiment.

### In Vivo Experiments

#### Animals

Male adult CD1 mice, 6 weeks old (25–30 g, Envigo, Udine, Italy), were properly housed and provided with standard rodent chow and water in steel cages at room kept at 22 ± 1 °C with a 12-h light, 12-h dark cycle. The animals were accustomed ad libitum access to tap water and a standard rodent diet. This study was approved by the University of Messina Review Board for the care of animals, in compliance with Italian regulations on the protection of animals (n° 399/2019-PR released on 05/24/2019). Animal care was performed in accordance with Italian regulations on the use of animals for the experiment (D.M. 116192) as well as with EEC regulations (O.J. of E.C. L 358/1 12/18/1986). The animal protocol was declared exempt, by an institutional review board [University of Messina Review Board for the care of animals, in compliance with Italian regulations on the protection of animals (n° 399/2019-PR released on 05/24/2019)] in 2016 and confirmed in 2019.

#### Surgical Procedure for Spinal Cord Injury

SCI was performed as previously described (Paterniti et al. 2018; Filippone et al. 2020). Briefly, mice were anesthetized with intraperitoneal (i.p.) xylazine and ketamine (0.16 and 2.6 mg/kg body weight, respectively). Mice were subsequently stabilized, and the spinal cords were exposed via laminectomy. The SCI procedure was reproduced by extradural compression of the spinal cord using an aneurysm clip with a closing force of 24 g along the thoracic vertebra 6–7 (T6–T7) for 1 min. During post-surgery, the mice were placed on a warm heating pad and covered with a warm towel, and the bladder of the mice was manually emptied at intervals (every 2 h) immediately after waking up mice from anesthesia (Casili et al. 2020). Animals were euthanized 24 h after trauma induction.

#### Experimental Groups

Mice were allocated into the following groups:

- Sham + vehicle: mice were subjected to laminectomy, but the aneurysm clip was not applied, and the mice were treated with vehicle (saline i.p., 30 min after laminectomy) (*n* = 10).
- SCI + vehicle: mice were subjected to SCI and treated with saline (i.p., 30 min after SCI) (*n* = 10).
- SCI + trehalose: mice were subjected to SCI, and trehalose was administered (i.p., at a dose of 150 mg/kg) 1 h and 6 h after SCI (*n* = 10).
- SCI + carnosine: mice were subjected to SCI, and carnosine was administered (i.p., at a dose of 150 mg/kg) 1 h and 6 h after SCI (*n* = 10).
- SCI + conjugate: mice were subjected to SCI, and the conjugate was administered (i.p., at a dose of 150 mg/kg) 1 h and 6 h after SCI (*n* = 10).

The dose of 150 mg/kg has been selected and used on the basis of the previous studies that reported administration of this dose intraperitoneally at 1 and 6 h after SCI (Albayrak et al. 2015; Di Paola et al. 2011; Stvolinsky et al. 2017). Moreover, treatments were administered 1 and 6 h post-injury since these time points reflect clinically relevant and effective time (therapeutic window) for counteracting acute damage.

Mice were divided following simple randomization and partial blinding methods as previously described (Bespalov et al. 2019). Moreover, the authors were blinded while performing all the experiments. The minimum number of mice for every technique was estimated with the statistical test “ANOVA: Fixed effect, omnibus one-way” with G-power software. This statistical test generated a sample size equal to *n* = 10 mice for each technique.

#### Histological Examination

Spinal cord tissues were collected 24 h after treatment. After fixing the tissues in buffered formaldehyde solution (10% in phosphate-buffered saline (PBS), sagittal sections were prepared and stained with hematoxylin and eosin (H&E) as previously described (Casili et al. 2020) and evaluated using a Leica DM6 microscope (Leica Microsystems SpA, Milan, Italy) associated with Leica LAS X Navigator software using the objective lens at 10 × magnification (Leica Microsystems SpA, Milan, Italy). The following morphological criteria were considered: (1) No pathological abnormalities; (2) Small, focal, scattered areas of axonal swelling; morphologically unremarkable tissue in > 75% of the perilesional area; (3) Significant diffuse damage with normal gross architecture; unremarkable tissue in 50–75% of the perilesional area; (4) Significant diffuse damage with normal gross architecture; morphologically unremarkable tissue in 25–50% of perilesional area; (5) Significant diffuse damage and loss of gross architecture in large areas; morphologically unremarkable tissue in 10–25% of perilesional area; (6) Complete dissolution of the spinal cord over the entire cross-sectional area with loss of gross architecture; morphologically unremarkable tissue in < 10% of perilesional area. The results from every section of the spinal cord were averaged to obtain a final score (1 to 5) for distinct mice.

#### Western Blot Analysis for IkB-α NF-kB, p-Akt, PI3K, p-CREB, p-ERK, Bax, Bcl-2, p53, Caspase-3, BDNF, GDNF and Zn Transporters

Spinal cord tissue from each mouse was suspended in extraction buffer A containing 0.2 mM PMSF, 0.15 mM pepstatin A, 20 mM leupeptin, 1 mM sodium orthovanadate, homogenized at the maximum setting for 2 min, and centrifuged at 12,000 × rpm for 4 min at 4 °C. Supernatants represented the cytosolic fraction. The pellets, containing enriched nuclei, were resuspended in buffer B containing 1% Triton X-100, 150 mM NaCl, 10 mM Tris–HCl pH 7.4, 1 mM EGTA, 1 mM EDTA, 0.2 mM PMSF, 20 mM leupeptin, 0.2 mM sodium orthovanadate. After centrifugation for 10 min at 12,000 rpm at 4 °C, the supernatants containing the nuclear protein were stored at − 80 °C for further analysis. Proteins from cytoplasm and nuclear fraction were added to sample buffer (0.125 M Tris–HCl, (pH 6.8), 4% SDS, 20% glycerol, 10% β-mercaptoethanol, 0.004% bromophenol blue), and boiled in a water bath for 5 min. Protein samples were separated on denatured 12% SDS polyacrylamide gel and transferred to a nitrocellulose membrane. Non-specific binding to the membrane was blocked for 1 h at room temperature with 5% non-fat dry milk (PM) in PBS. Membranes were incubated at 4 °C overnight with primary antibodies in milk–PBS–Tween 20, 0.1% (PMT). Levels of nuclear factor kappa-light-chain-enhancer of activated B cells (NF-*k*B), (1:100, sc8008, Santa Cruz Biotechnology, Dallas, TX USA) nuclear factor of kappa light polypeptide gene enhancer in B-cells inhibitor, alpha (I*k*B-α) (1:100, sc1643, Santa Cruz Biotechnology, Dallas, TX USA), phospho-RAC-alpha serine/threonine-protein kinase (p-Akt) (1:1000, Cell Signaling Technology, CST 9275), phospho-cAMP response element-binding protein (p-CREB) (1:500; Santa Cruz Biotechnology, sc-81486), phosphoinositide 3-kinase (PI3K), extracellular signal-regulated kinase (p-ERK), Bax (1:500 sc-7480, Santa Cruz Biotechnology, Dallas Texas TX USA), Bcl-2 (1:500 sc-7382 Santa Cruz Biotechnology, Dallas Texas TX USA), p53 (1:500 sc-98, Santa Cruz Biotechnology, Dallas Texas TX USA), Caspase-3 (1:500 sc-7272, Santa Cruz Biotechnology, Dallas Texas TX USA), brain-derived nerve factor (BDNF) (1:500 sc 20981; Santa Cruz Biotechnology, Dallas Texas TX USA), glial cell-derived nerve factor (GDNF) (1:500 sc-328; Santa Cruz Biotechnology, dallas Texas TX USA), Zn transporters (ZnT1), (1:1000 ARP44019), Aviva Systems Biology (CA, USA) and ZnT3 (1:1000 ARP43848); Aviva Systems Biology (CA, USA) were quantified in spinal cord tissue collected after 24 h after SCI. Membranes were blocked in 5% (w/v) non-fat dried milk in buffered saline (PM) for 45 min at room temperature and subsequently probed with specific antibodies listed above, in 1 × PBS, 5% w/v non-fat dried milk, and 0.1% Tween-20 (PMT) at 4 °C overnight. Membranes were incubated with peroxidase-conjugated bovine anti-mouse immunoglobulin G (IgG) or peroxidase-conjugated goat anti-rabbit IgG secondary antibody (1:2000, #AB2307391 (rabbit) #AB10015289 (mouse), Jackson ImmunoResearch, West Grove, PA) for 1 h at room temperature. To determine whether blots were loaded with equal amounts of proteins, they were also incubated in the presence of antibodies against β-actin protein (cytosolic fraction 1:500; sc-8432Santa Cruz Biotechnology, Dallas Texas TX USA), or laminin A/C fraction (1:500; sc-74418 Santa Cruz Biotechnology Dallas Texas TX USA), Akt antibody (1:500, Cell Signalling, #9272), CREB (Biotech, Life Sciences, ab-32515) or ERK1/2 (1:1000, Cell Signalling Technology, CST 5627S). Signals were detected using enhanced chemiluminescence (ECL) detection system reagent according to the manufacturer’s instructions (Thermo, USA). The relative expression of the protein bands was quantified by densitometry using BIORAD ChemiDoc TMXRS + software and standardized to β-actin levels as an internal control. We validated all of the used antibodies choicing and preparing cell lines or tissue samples, consisting of true positive and negative controls.

#### Immunolocalization of Nitrotyrosine, Poly-(ADP-ribosio)-polymerase (PARP), Bcl-2 and Bax by Immunohistochemistry (IHC)

Sagittal spinal cord sections were deparaffinized and rehydrated as previously described (Lanza et al. 2019) Then, the sections were incubated overnight (O/N) with primary nitrotyrosine (Santa Cruz Biotechnology Dallas Texas TX USA; sc32757, 1:100 in PBS), poly-(ADP-ribosio)-polymerasi (PARP) (Santa Cruz Biotechnology; Dallas Texas TX USA, sc8007, 1:100 in PBS), Bcl-2 Santa Cruz Biotechnology; 1:100 in PBS), and Bax (Santa Cruz Biotechnology; 1:100 in PBS). Sections were washed with PBS and incubated with peroxidase-conjugated bovine anti-mouse immunoglobulin G (IgG) or peroxidase-conjugated goat anti-rabbit IgG secondary antibody (1:2.000 Jackson Immuno Research, West Grove, PA, USA). Specific labelling was detected using a biotin-conjugated goat anti-rabbit IgG or biotin-conjugated goat anti-mouse IgG and avidin–biotin peroxidase complex (Vector Laboratories, Burlingame, CA, USA). Immunohistochemical images were obtained and observed using a Zeiss microscope with Axio Vision software. The percentage area of immunoreactivity (brown staining, determined by the number of positive cells) is expressed as % of the total tissue area (red staining) of five random fields with objective lens at 20 × magnification, and the analysis was performed using ImageJ. Densitometry analysis was performed using GraphPad version 5.0 (La Jolla, CA, USA).

#### Immunofluorescence Staining for BDNF and GDNF

Sagittal spinal cord sections were processed for immunofluorescence staining as previously described (Campolo et al. 2020). Sections were incubated with anti-BDNF (1:100) or anti-GDNF (1:100) antibody in a humidified chamber O/N at 37 °C. Sections were then incubated with Texas Red-conjugated anti-rabbit Alexa Fluor-594 secondary antibody (#A11037 1:1000 in PBS, vol/vol Molecular Probes, Monza, Italy) for 1 h at 37 °C. Nuclei were stained by adding 2 μg/ml 4′,6′-diamidino-2-phenylindole (DAPI; #5748, Hoechst, Frankfurt, Germany) in PBS. Sections were observed with an objective lens at 40 × magnification using a Leica DM2000 microscope (Leica, Milan, Italy). Contrast and brightness were established by examining the most brightly labeled pixels and applying settings that allowed clear visualization of structural details while keeping the highest pixel intensities close to 200. The same settings were used for all images obtained from the other samples that had been processed in parallel.

#### Detection of 8-Hydroxy-2′-deoxyguanosine (8-OHdG) Content

ELISA method (DNA damage competitive ELISA Kit #EIADNAD) was used to detect the content of 8-OhdG in the serum of mice at 24 h.

### Statistical Evaluation

All values in the figures and text are expressed as ± SD. For the in vivo studies, N represents the number of animals studied. The three experiments performed on different days stand for three biological replicates. The Shapiro–Wilk test was used for the normality distribution analysis. The results were analysed by one-way ANOVA followed by a Bonferroni post hoc test for multiple comparisons. A p value of less than 0.05 was considered significant.

## Results

### Synthesis of Trehalose Conjugate with Carnosine

#### Tre–Car is Obtained Through a Revised Synthetic Route

Although the synthesis of Tre–car (1) has been previously described in the literature (Grasso et al. 2011; Rizzarelli et al. 2007) we chose to follow the synthetic route shown in Scheme 1 because this pathway is better suited the needs of preparing the conjugate in sufficient amounts for its subsequent use in in vivo assays. 6-Bromo-6-deoxy-α,α-trehalose was prepared by bromination of trehalose, and then, the nucleophilic substitution of the halogen by the amine group of carnosine led to the formation of the desired conjugate.

### In Vitro Experiments

The homeostasis of intracellular Zn^2+^ is strongly regulated and although the major fraction of the metal ion is tightly bound, functioning as a catalytic or structural component of proteins, a loosely bound minor fraction, termed labile or exchangeable zinc, modulates the activity of numerous signaling and metabolic pathways. Specific fluorophores with high affinity for zinc have been employed to detect this metal ion pool to monitor cellular Zn^2+^ dynamics in situ (Gee et al. 2002), also unveiling the ionophore ability of different zinc ligands (Dabbagh-Bazarbachi et al. 2014). As above cited, transmembrane zinc transporters control the cellular Zn^2+^ uptake and efflux; among the zinc export transporters, the ZnT1 (Shusterman et al. 2014), located at the plasma membrane, is the main regulator of excess zinc cell export. In this context, the zinc probe Fluo-Zin-3 (Gee et al. 2002) was used to assign the ionophore ability of carnosine and its conjugate with trehalose, while the ZnT1 allowed us to follow the dynamics of zinc labile pool fluxes.3.1.2 Tre–car increases Zn^2+^ intracellular concentration.

#### Tre–Car Increases Zn^2+^ Intracellular Concentration

**Fig. 1:**
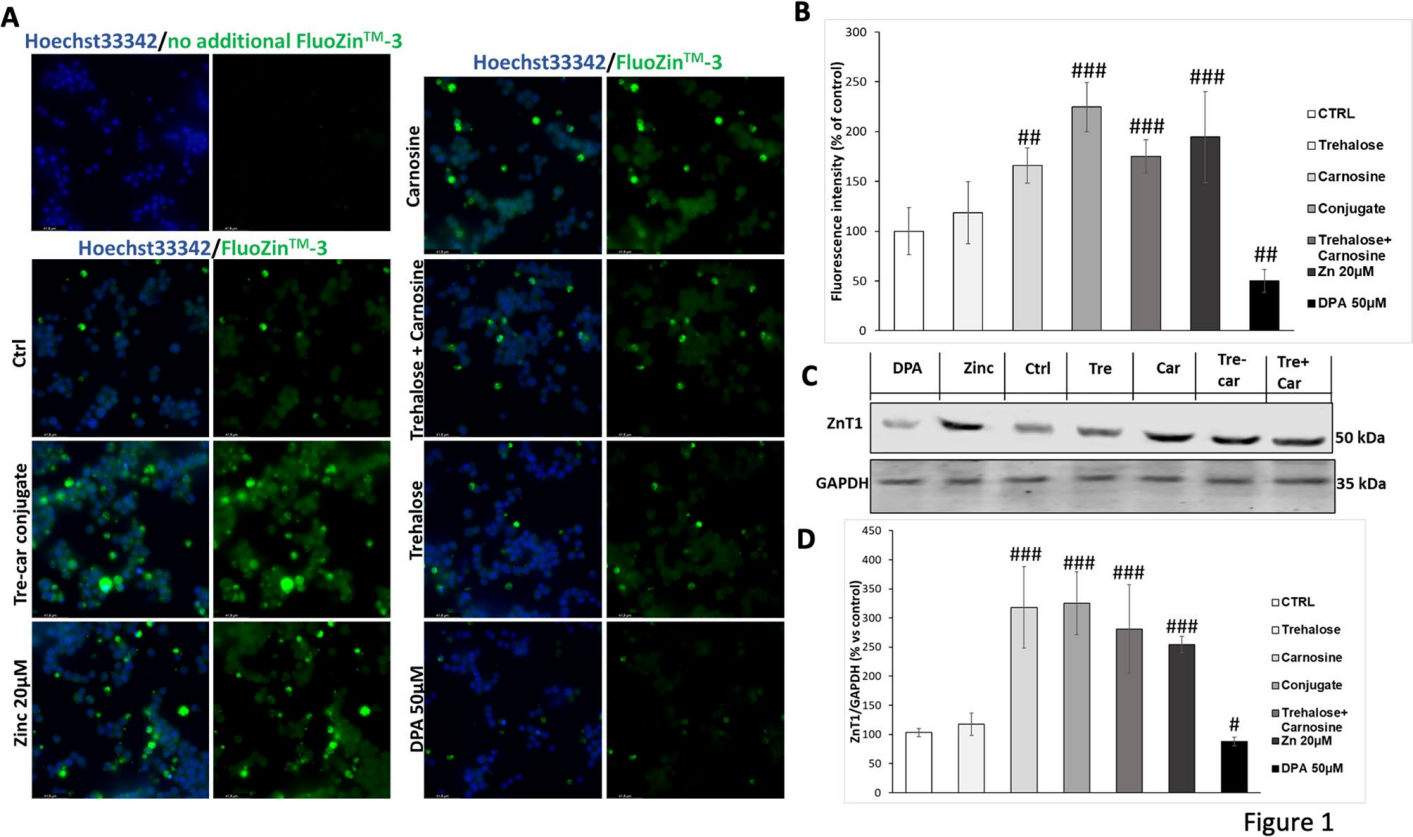
**A**, **B** Effects of Tre–car on the cytoplasmic pool of labile zinc in PC12 cells. Average intensity values for the FluoZin-3 emission corresponding to the Zn^2+^ content in the cytoplasm for control untreated cells and treated 20 h with Tre, Car, Tre–car or Tre + Car mixture in RPMI1640 complete medium with 1% HS and 0.5% FBS; 20 µM zinc (Magri et al. 2016) or 50 µM membrane-impermeable zinc chelator DPA treatment were used as a positive and negative control, respectively. The effect of Tre–car, Car or Tre + Car mixture treatment analysed by fluorescence images of cells incubated with zinc probe (**A**) and quantification of fluorescent intensity normalized to the number of cells presented in each field (**B**) confirms the significantly increased zinc concentration in PC12 cells. As a baseline to exclude cell auto-fluorescence, PC12 cells were treated only with Hoechst33342 without FluoZin-3. Scale bars are 42 μm. All values are mean ± SD of three independent experiments of 6–8 randomly chosen fields. Significant differences between treatments were determined using one-way ANOVA method ^#^*p* ≤ 0.05, ^##^*p* ≤ 0.01, ^###^*p* ≤ 0.001 versus untreated control cells. **C** Tre, Car, Tre–car or Tre + Car mixture affected ZnT1 expression. Starved PC12 cells were stimulated for 24 h with 5 mM Tre, Car, Tre–car, Tre + Car, 20 µM zinc or 50 µM membrane-impermeable zinc chelator DPA in RPMI11640 complete medium with 1% HS and 0.5% FBS. The expression level of ZnT1 is reported as a ratio to that of GAPDH. Treatment with Car, Tre–car or Tre + Car mixture significantly increased ZnT1 expression. All values are mean ± SD ^#^*p* < 0.01, ^##^*p* < 0.001 versus untreated control cells (samples *n* = 4, three individual experiments)

Fluorescent microscopy imaging studies of PC12 cells stained with the zinc sensor FluoZin-3, clearly show an increase of the fluorescent signals related to zinc ions with respect to the control untreated cells, after treatment with 5 mM Tre–car (224.7.0% ± 24.7, ^###^*p* ≤ 0.001), Car (165.9% ± 17.5, ^##^*p* ≤ 0.01) or Tre + Car (175.0% ± 16.8, ^###^*p* ≤ 0.001), 20 µM Zn(II) alone (194.5% ± 45.7, ^###^*p* ≤ 0.001) and 50 μM DPA (49.9% ± 11.6, ^#^*p* ≤ 0.05) (Fig. 1a). As a baseline to exclude cell auto-fluorescence, PC12 cells were treated only with Hoechst33342 without FluoZin-3. The treatment with Tre do not appear to favour the cell uptake of Zn(II) after 20 h incubation whereas the presence of zinc chelator DPA significantly reduced the FluoZin-3 fluorescence level. This observation is confirmed by the quantitative analysis of the FluoZin-3 emission (Fig. 1b) over the whole cell area. The higher value measured for Tre–car treatment in comparison to Car and Tre + car mixture treated-cells is statistically significant. We may conclude that Car, its Tre derivate and mixture Tre with car cause Zn(II) translocation into cytoplasm from cell cultural medium; in this context, Tre–car is more effective than car alone or its mixture with Tre, in keeping with the major ionophore ability of conjugated molecule (Naletova et al. 2021).

#### Tre–Car Alters Zn^2+^ Homeostasis, Affecting ZnT1, the Membrane Efflux Transporter of Metal Ions and ZnT3

Since 1976, the PC12 cell line has been employed for investigating multiple aspects of neurobiology, including neuronal differentiation, intracellular signalling pathways, and cell survival (Greene and Tischler 1976; Hu et al. 2018). In the present study, PC12 cells were utilized to determine the effect of Tre–car on zinc transporter ZnT1 expression levels in vitro*.* This membrane transporter acts as a probe of intracellular zinc(II) ion levels and induces metal ion efflux to guarantee metallostasis (Wang et al. 2011b). Following immunoblot analysis (Fig. 1c, d) Normal Distribution: SPSS test, CTRL *W* = 0.8949, *p* = 0.406; Tre *W* = 0.8779, *p* = 0.330; Car *W* = 0.9432, *p* = 0.674; Tre–Car *W* = 0.9370, *p* = 0.636; Tre + Car *W* = 0.901, *p* = 0.436; *F*(4,15) = 43.3, *p* = 0.541467, one-way ANOVA method, followed by Bonferroni post hoc test for multiple comparisons), treatment with 5 mM carnosine, trehalose–carnosine or trehalose + carnosine mixture induced an increase in the expression levels of ZnT1 up to 318% ± 70, 326% ± 54 and 281% ± 76, respectively. This finding clearly indicates an ionophore ability of carnosine (also in the mixture with trehalose) and its conjugate due to their chelating features.

### In Vivo Experiments

#### The Tre–Car Conjugate Influences Zn^2+^ Homeostasis in Response to SCI

**Fig. 2:**
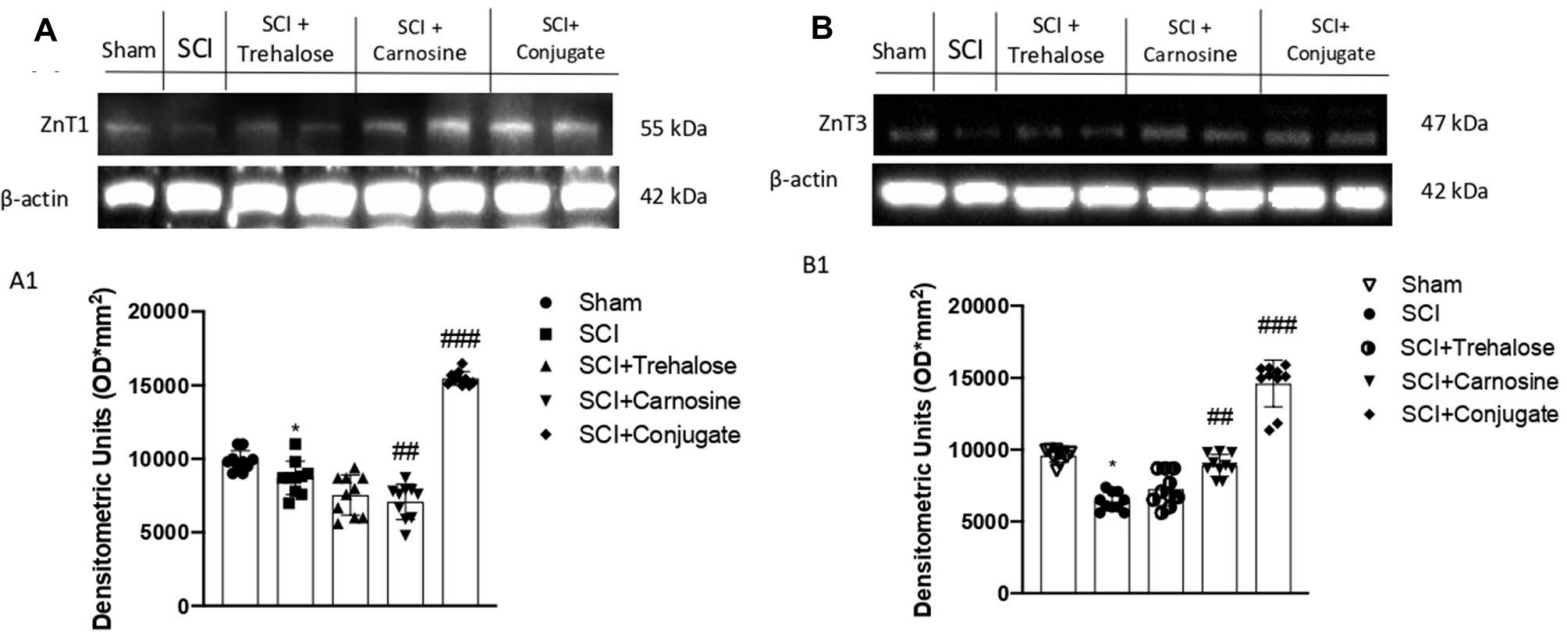
Tre, Car or Tre–car differentially affected ZnT1 and ZnT3 expression after SCI. The expression level of zinc transporters is reported as a ratio to actin. A representative blot of lysates is shown, and densitometry analysis is reported. Data are expressed as SD

To determine whether Tre–car affects Zn transporter 1 expression levels in a mouse model of SCI, rodents were treated with the different compounds (i.p., at a dose of 150 mg/kg), and ZnT1 expression was detected in tissue lysates. The results indicated that SCI induces a significant decrease in ZnT1 expression; conversely, treatment with Car and more so with Tre–car resulted in an increase in the expression levels of the membrane transporter (Fig. 2a; Normal Distribution: SPSS test, Sham *W* = 0.8869, *p* = 0.157; SCI *W* = 0.9394, *p* = 0.546; SCI + Trehalose *W* = 0.9027 *p* = 0.235; SCI + Carnosine *W* = 0.9136, *p* = 0.307; SCI + Conjugate *W* = 0.894, *p* = 0.188; *F*(4,45) = 2.33, *p* = 0.070292, one-way ANOVA method, followed by Bonferroni post hoc test for multiple comparisons).

This enhancement indicates that both the dipeptide and its derivative with trehalose favor the cellular uptake of zinc(II) ions, inducing the activation of the efflux process to counteract the excessive intracellular increase in the metal ion amount. Among the ZnT family components that decrease the cytosolic content of zinc(II) ions, ZnT3 performs this task by confining metal ions into synapses (Portbury et al. 2017). Different studies report both a decrease in Znt3 protein levels in the spinal cords of ALS patients (Kaneko et al. 2015) and a significant loss of synaptic vesicular zinc with a decline in ZnT3 transcriptional activity in Huntington's disease (HD) transgenic mice (Niu et al. 2020). As a consequence of disrupted vesicular zinc synapses, dysfunction and cognitive deficits occur in HD. Analogously, SCI induced a significant decrease in ZnT3 expression levels, while both Car and Tre–car counteracted this effect (Fig. 2b; Normal Distribution: SPSS test, *W* = 0.8890, Sham *p* = 0.165; SCI *W* = 0.9171, *p* = 0.334; SCI + Trehalose *W* = 0.9017, *p* = 0.229; SCI + Carnosine *W* = 0.8944, *p* = 0.190; SCI + Conjugate *W* = 0.9320, *p* = 0.468; *F*(4,45) = 1.3, *p* = 0.002992, one-way ANOVA method, followed by Bonferroni post hoc test for multiple comparisons). Overall, the carnosine conjugate showed a protective effect against the alteration of certain ZnT transporters induced by SCI.

#### Tre–Car Attenuates the Severity of Spinal Cord Trauma, Decreasing Tissue Damage

**Fig. 3:**
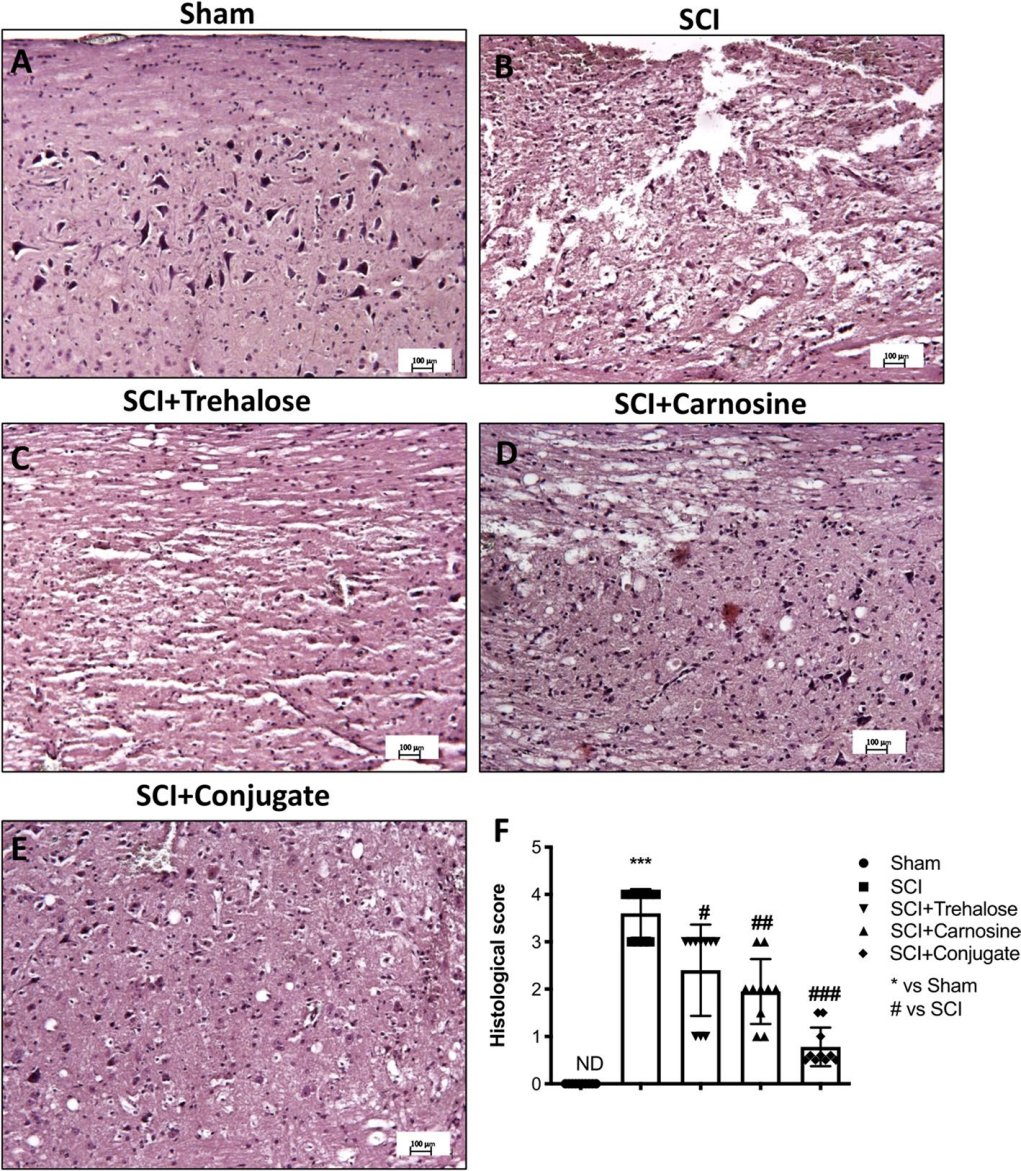
The severity of tissue damage following SCI is decreased in Tre–car-treated mice. Extensive damage to the spinal cord was observed in the SCI mouse group (**B**) compared to the sham mouse group (**A**). **C**, **D** Tre and Car treatments after SCI. **E** Tre–car treatment significantly reduces the SCI lesion score. **F** Relative histological score. ***p < 0.001 versus sham group; ^#^*p* < 0.05 versus SCI group; ^##^P < 0.01 versus SCI group. ^###^*p* < 0.001 versus SCI group. (samples *n* = 10, three individual experiment). Data are expressed as SD

The first step of SCI consists of immediate mechanical injury that causes loss of tissue architecture and recruitment of proinflammatory mediators at the damaged site. The longitudinal sections of the spinal cord were used to perform H&E staining to observe the potential effects of the Tre–car conjugate after injury. Significant structural changes occurred in the spinal cord of injured mice (Fig. 3b) compared to sham mice (Fig. 3a) with respect to the loss of tissue architecture, oedema, and accumulation of neutrophils, which are major proinflammatory signs that appear after SCI. The severity of the injury decreased with Tre (Fig. 3c) and Car treatment alone (Fig. 3d, see histological score F; Normal Distribution: SPSS test, Sham ND; SCI *W* = 0.8707, *p* = 0.102; SCI + Trehalose *W* = 0.8658, *p* = 0.089; SCI + Carnosine *W* = 0.8827, *p* = 0.140; SCI + Conjugate *W* = 0.8946, *p* = 0.191; *F*(4,36) = 69.46, *p* = 0.026392, one-way ANOVA method, followed by Bonferroni post hoc test for multiple comparisons), showing fewer dead and degenerated neurons compared to injured mice. However, Tre–car showed a significant ability to repair damaged tissue (Fig. 3, Panel E, see histological score in F), suggesting the successful conjugation of Car with Tre in the attenuation of neuronal degeneration.

#### The Tre–Car Conjugate Decreases Activation of the Inflammatory Cascade Induced by SCI

**Fig. 4:**
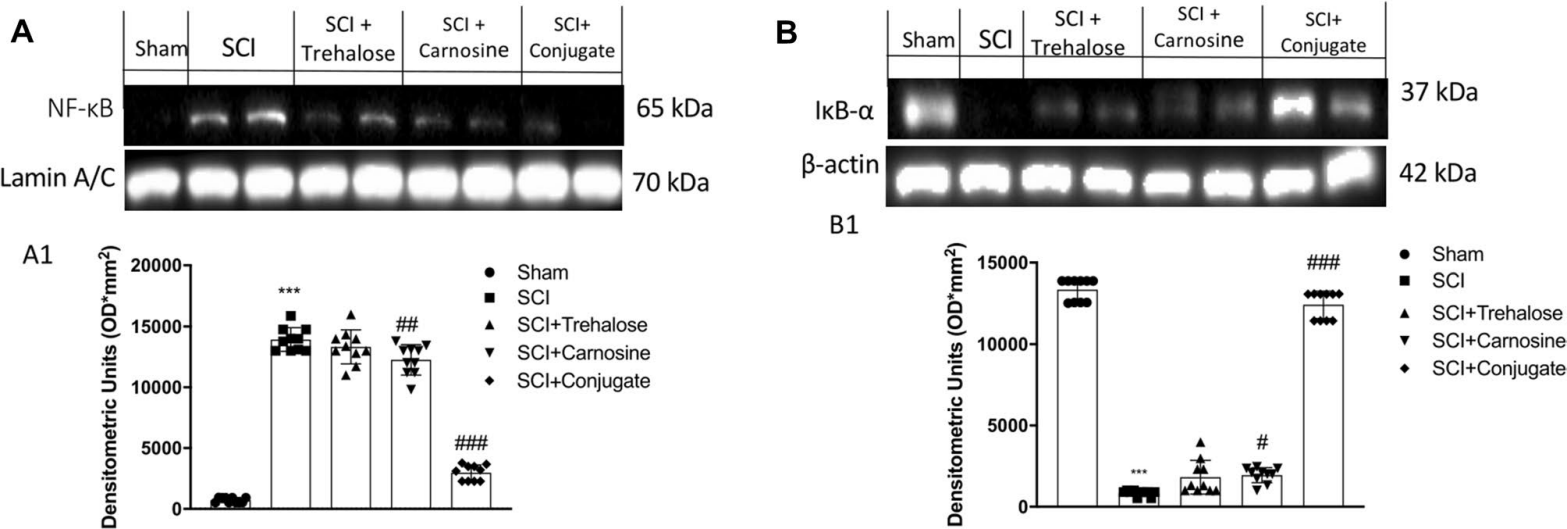
Western blot analysis of spinal cord samples. **A** NF-kB expression level evaluation and relative densitometric analysis shown in A1. **B** IκB-α expression level evaluation and relative densitometric analysis shown in B1. ***p < 0.001 versus sham group; ^#^*p* < 0.05 versus SCI group; ^##^*p* < 0.01 versus SCI group. ^###^*p* < 0.001 versus SCI group (samples *n* = 10, three individual experiments)

To gain insights into the mechanisms underlying the effects of Tre–car on acute effect in inflammation, we evaluated the expression levels of nuclear factor NF-kB and its inhibitor IκB-α. Nuclear translocation of NF-kB was higher in the SCI group than in the sham group, while treatment of mice with Tre–car remarkably decreased NF-kB translocation (Fig. 4, panel A, see densitometric analysis Panel A1;Normal Distribution: SPSS test, Sham *W* = 0.8460, *p* = 0.052; SCI *W* = 0.8759, *p* = 0.117; SCI + Trehalose *W* = 0.9696, *p* = 0.887; SCI + Carnosine *W* = 0.9269, *p* = 0.418; SCI + Conjugate *W* = 0.8452, *p* = 0.051; *F*(4,45) = 3.48, *p* = 0.014710, one-way ANOVA method, followed by Bonferroni post hoc test for multiple comparisons). Different changes occur after treatment with Car and Tre; the disaccharide shows a lesser ability to counteract the nuclear translocation of NF-kB than the dipeptide, highlighting the advantage of the conjugate treatment. SCI mice exhibit increased degradation of IκB-α compared to the Sham mice; the conjugate successfully decreases the degradative process, differently from the treatment with Tre and Car that slightly modify the process (Fig. 4b, see densitometric analysis in B1; Normal Distribution: SPSS test, Sham *W* = 0.8531, *p* = 0.063; SCI *W* = 0.8497, *p* = 0.058; SCI + Trehalose *W* = 0.8466, *p* = 0.053; SCI + Carnosine *W* = 0.8885, *p* = 0.163; SCI + Conjugate *W* = 0.8786, *p* = 0.126; *F*(4,45) = 1.75, *p* = 0.155842, one-way ANOVA method, followed by Bonferroni post hoc test for multiple comparisons).

#### Tre–Car Attenuates the Oxidative Stress Induced by SCI

**Fig. 5:**
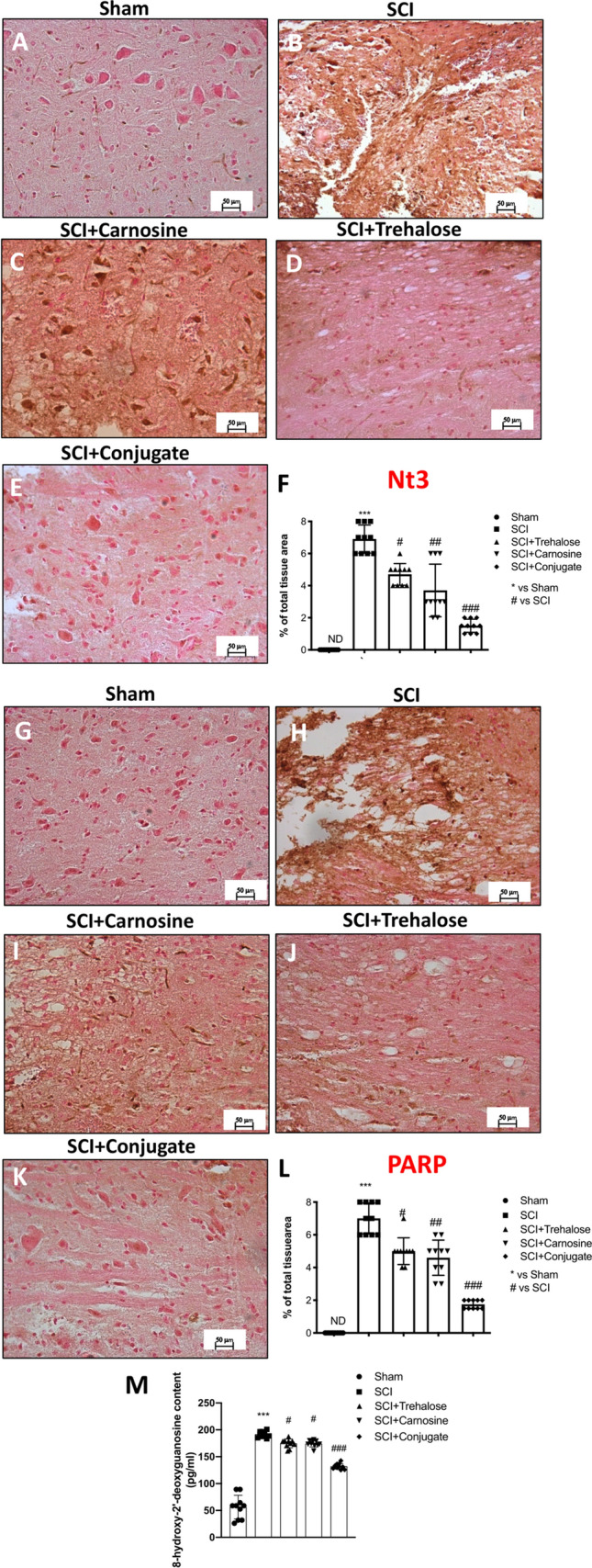
Effects of Tre–car on Nt and PARP. A substantial increase in Nt-positive staining was observed in spinal cord tissues collected from mice 24 h after SCI compared to sham mice (**B**, **A**). Treatment with Tre and Car reduced the positive staining of Nt (**C**, **D**). Tre–car treatment protects tissue after SCI (**E**). A substantial increase in PAR-positive staining was observed in spinal cord tissues of SCI mice compared to sham mice (**H**, **G**). Treatment with Tre and Car reduced the positive staining of PARP (**I**, **J**). Tre–car treatment protects tissue after SCI (**K**). Quantitative panels of Nt and PARP, respectively (**F**, **L**). 8-hydroxy-2-deoxyguanosine (8-OHdG) content in serum (M). ***p < 0.001 versus sham; ^#^*p* < 0.05 versus SCI; ^##^*p* < 0.01 versus SCI; ^###^*p* < 0.001 versus SCI; ND not detectable. (samples *n* = 10, three individual experiments). Data are expressed as SD

Hypoxia–ischaemia and consequent early initiated inflammation in SCI include various events, such as the production of excitatory amino acids, altered ion homeostasis, induction of oxidative stress and ROS and RNS production, contributing to neuronal cell death (Jia et al. 2012) by forming the free radical superoxide and peroxynitrite, which damage fatty acids, lipids, proteins and DNA (Chen et al. 2018a; Stewart et al. 2021). Thus, we examined whether Tre–car could alleviate the oxidative stress induced by SCI. Using nitrotyrosine (Nt) as a marker of oxidative stress, we found that nitrotyrosine immunoreactivity was markedly increased after SCI compared to the sham group (Fig. 5). In contrast, the conjugate affected Nt immunoreactivity, which resulted in significantly decreased levels (Fig. 5e) compared to the minor effect of Tre (Fig. 5c) or Car (Fig. 5d) alone (% of Nt area panel F; Normal Distribution: SPSS test, Sham ND; SCI *W* = 0.8709, *p* = 0.102; SCI + Trehalose *W* = 0.8551, *p* = 0.067; SCI + Carnosine *W* = 0.8721, *p* = 0.106; SCI + Conjugate *W* = 0.8709, *p* = 0.102; *F*(4,36) = 78.94, *p* = 0.927336), one-way ANOVA method, followed by Bonferroni post hoc test for multiple comparisons). The ability of Tre–car to alleviate SCI-induced oxidative stress in spinal cord tissues was further supported by PARP immunoreactivity results that clearly indicated the conjugate prevents DNA damage induced by SCI. No immunopositive neurons were found in the spinal cord tissues of the sham mice (Fig. 5g), whereas increased immunoreactivity of PARP was evident in SCI mice (Fig. 5h). In Tre- (Fig. 5i) and Car-treated (Fig. 5j) mice, PARP immunoreactivity was attenuated, while the conjugate consistently reduced immunoreactivity within 24 h after SCI (Fig. 5k) (% of PARP area panel L; Normal Distribution: SPSS test, Sham ND; SCI *W* = 0.8553, *p* = 0.067; SCI + Trehalose *W* = 0.8764, *p* = 0.119; SCI + Carnosine *W* = 0.8917, *p* = 0.177; SCI + Conjugate *W* = 0.8774, *p* = 0.122; *F*(4,45) = 4.752, *p* = 0.002769, one-way ANOVA method, followed by Bonferroni post hoc test for multiple comparisons). Moreover, we examined a presence of 8-hydroxy-2-deoxyguanosine (8-OHdG), one of the best markers of the oxidative DNA damage, in the spinal cord of mice. Results showed that the levels of protein 8-OHdG in control mice (Sham group) expressed a little. This oxidative stress indicator showed obvious increase in SCI-injured mice (SCI group) when compared to the control mice. Trehalose and Carnosine treatments (SCI + Trehalose group) (SCI + Carnosine group) attenuated SCI-induced DNA damage in a significant manner. Particularly, Conjugate treatment showed greater ability to significantly attenuate DNA damage after SCI (Fig. 5m; Normal Distribution: SPSS test, Sham *W* = 0.9034, *p* = 0.238; SCI *W* = 0.9629, *p* = 0.818; SCI + Trehalose *W* = 0.9347, *p* = 0.495; SCI + Carnosine *W* = 0.8968, *p* = 0.202; SCI + Conjugate *W* = 0.9571, *p* = 0.752; *F*(4,45) = 4.60;p.003; one-way ANOVA method, followed by Bonferroni post hoc test for multiple comparisons).

#### Tre–Car Displays Anti-apoptotic Ability

**Fig. 6:**
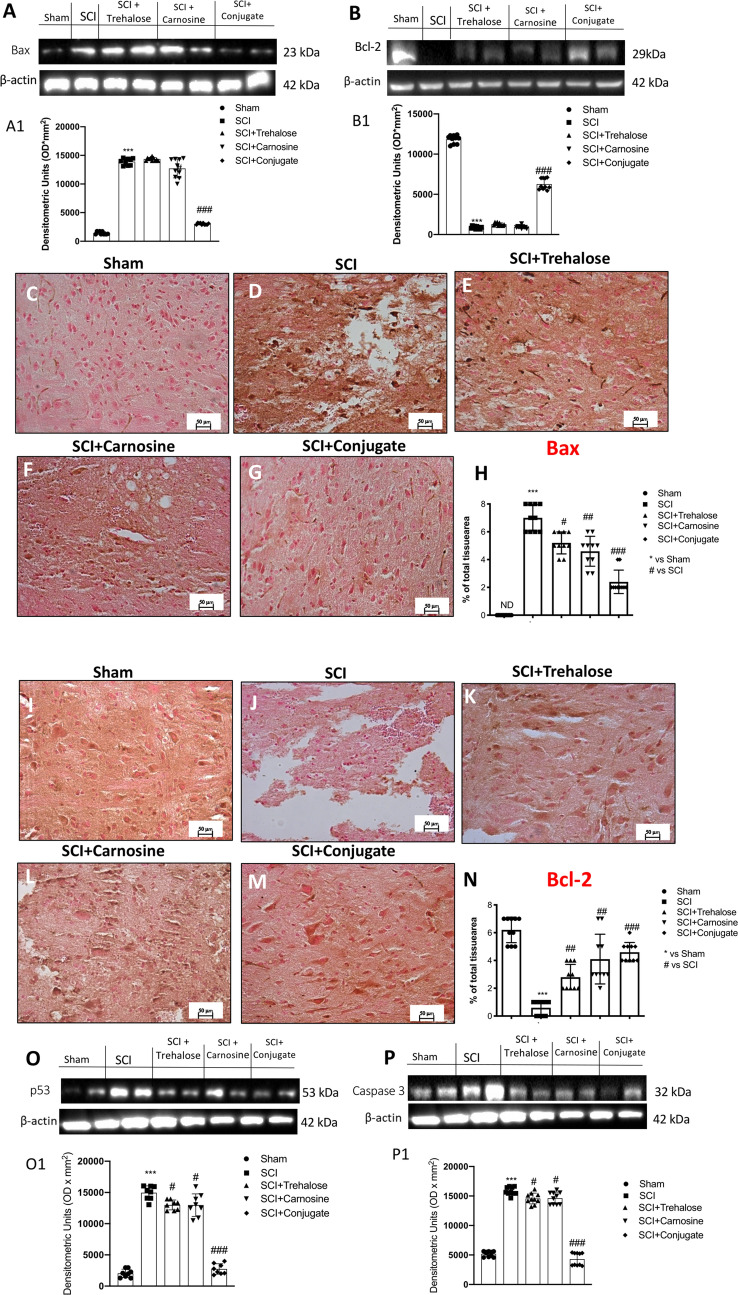
Effects of Tre–car on apoptosis levels. Western blot analysis showing the expression of Bax (**A**) and Bcl-2 (**B**) 24 h after SCI with densitometric analysis shown A1 and B1. A substantial increase in Bax-positive staining was observed in spinal cord tissues collected from mice 24 h after SCI compared to sham mice (**D**, **C**) (samples *n* = 10, three individual experiments). Treatment with Tre and Car reduced the positive staining of Bax (**E**, **F**). Tre–car treatment protects tissues after SCI (**G**). A substantial increase in Bcl-2-positive staining was observed in sham mouse spinal cord tissues compared to SCI mice (**I**, **J**). Treatment with Tre and Car increased the positive staining of Bcl-2 (**K**, **L**). Tre–car treatment protects after SCI (**M**). Quantitative panels of Bax and Bcl-2 (**H**, **N**). p53 and Caspase-3 protein expression increased 24 h post SCI, compared with sham group, while Tre, Car and Conjugate treatments significantly both p53 and Caspase-3 expression levels (**O**, **P**, see densitometric analysis O1 and P1). ***p < 0.001 versus sham, ^#^*p* < 0.05 versus SCI; ^##^*p* < 0.01 and ^###^*p* < 0.001 versus SCI. ND not detectable. (samples *n* = 10, three individual experiments). Data are expressed as SD

Spinal cord trauma leads to the development of pathophysiological processes, including neuronal cell apoptosis and acute inflammation that affect the injured tissue, seriously compromising the conductive function of the nerves (Li et al. 2019c). Thus, we next determined whether Tre–car, Tre and Car mediate protective effects by inhibiting apoptosis by evaluating Bax and Bcl-2 expression, both proteins essential for apoptosis. First, the elevation of apoptosis induced by SCI was significantly attenuated by treatment with Tre–car, as estimated by protein expression level quantitation of the pro-apoptotic Bax (Kotipatruni et al. 2011) (Fig. 6a, see densitometric analysis in A1; Normal Distribution: SPSS test, Sham *W* = 0.8557, *p* = 0.068; SCI *W* = 0.8490, *p* = 0.057; SCI + Trehalose *W* = 0.8757, *p* = 0.116; SCI + Carnosine *W* = 0.8922, *p* = 0.180; SCI + Conjugate *W* = 0.8482, *p* = 0.055; *F*(4,45) = 20.2, *p* = 0.000001, one-way ANOVA method, followed by Bonferroni post hoc test for multiple comparisons). The mechanism by which the conjugate affects neuronal cell death was next analyzed by examining levels of antiapoptotic Bcl-2 after SCI. The Bcl-2 overexpression found in sham mice was significantly restored by administration of Tre–car, which protected neuronal cells from apoptosis induced by spinal cord trauma (Fig. 6b, see densitometric analysis in B1; Normal Distribution: SPSS test, Sham *W* = 0.8536, *p* = 0.064; SCI *W* = 0.9120, *p* = 0.295; SCI + Trehalose *W* = 0.8469, *p* = 0.053; SCI + Carnosine *W* = 0.8773, *p* = 0.122; SCI + Conjugate *W* = 0.8793, *p* = 0.128; *F*(4,45) = 4.87, *p* = 0.002380, one-way ANOVA method, followed by Bonferroni post hoc test for multiple comparisons). However, individual treatment with Tre or Car showed minimal antiapoptotic effects. Immunohistochemical analysis of spinal cord sections for both Bax and Bcl-2 was also performed to further confirm the protective effect of Tre–car in vivo. Bax immunoreactivity was primarily high in neurons of SCI mice (Fig. 6d) compared to sham mice (Fig. 6c). Although some neurons were positive in Tre- (Fig. 6e) or Car-treated (Fig. 6f) mice, there were significantly fewer Bax-positive cells in Tre–car-treated mice than in injured mice (Fig. 6g) (% of Bax area panel H; Normal Distribution: SPSS test, Sham ND; SCI *W* = 0.8737, *p* = 0.111; SCI + Trehalose *W* = 0.8729, *p* = 0.108; SCI + Carnosine *W* = 0.8917, *p* = 0.177; SCI + Conjugate *W* = 0.8456, *p* = 0.051; *F*(4,45) = 3.150, *p* = 0.022921, one-way ANOVA method, followed by Bonferroni post hoc test for multiple comparisons). The opposite results were found in Bcl-2 immunodetection due to the antiapoptotic role played by Tre–car administration after SCI (panels I to M) (% of Bcl-2 area panel N; Normal Distribution: SPSS test, Sham *W* = 0.8737, *p* = 0.111; SCI *W* = 0.8482, *p* = 0.055; SCI + Trehalose *W* = 0.8737, *p* = 0.111; SCI + Carnosine *W* = 0.8996, *p* = 0.217; SCI + Conjugate *W* = 0.8704, *p* = 0.101; *F*(4,45) = 1.595, *p* = 0.192104, one-way ANOVA method, followed by Bonferroni post hoc test for multiple comparisons). Moreover, the positive feedback in p53 accumulation to induce Caspase-3 activation was evaluated, and results showed that the expression levels of p53 and Caspase-3 significantly increased 24 h after SCI compared to the sham group, while Tre–Car conjugate treatment significantly reduced p53 and Caspase-3 expression levels despite moderate attenuation showed by Tre and Car treatments alone (Fig. 6o, p, see densitometric analysis O1 and P1; Normal Distribution: SPSS test, Sham *W* = 0.8999, *p* = 0.288; SCI *W* = 0.8883, *p* = 0.225; SCI + Trehalose *W* = 0.8767, *p* = 0.175; SCI + Carnosine *W* = 0.9704, *p* = 0.901; SCI + Conjugate *W* = 0.9135 *p* = 0.379. p53: *F*(4,35) = 2.13, *p* = 0.06, one-way ANOVA method, followed by Bonferroni post hoc test for multiple comparisons; Caspase 3: Normal Distribution: SPSS test, Sham *W* = 0.8697, *p* = 0.099; SCI *W* = 0.8861, *p* = 0.153; SCI + Trehalose *W* = 0.9688, *p* = 0.879; SCI + Carnosine *W* = 0.8947, *p* = 0.191; SCI + Conjugate *W* = 0.8530, *p* = 0.063; *F*(4,45) = 3,38;p.004; one-way ANOVA method, followed by Bonferroni post hoc test for multiple comparisons).

#### Modulation of the PI3K/Akt Pathway, p-ERK 1/2 and p-CREB is Mediated by Tre–Car Treatment

**Fig. 7:**
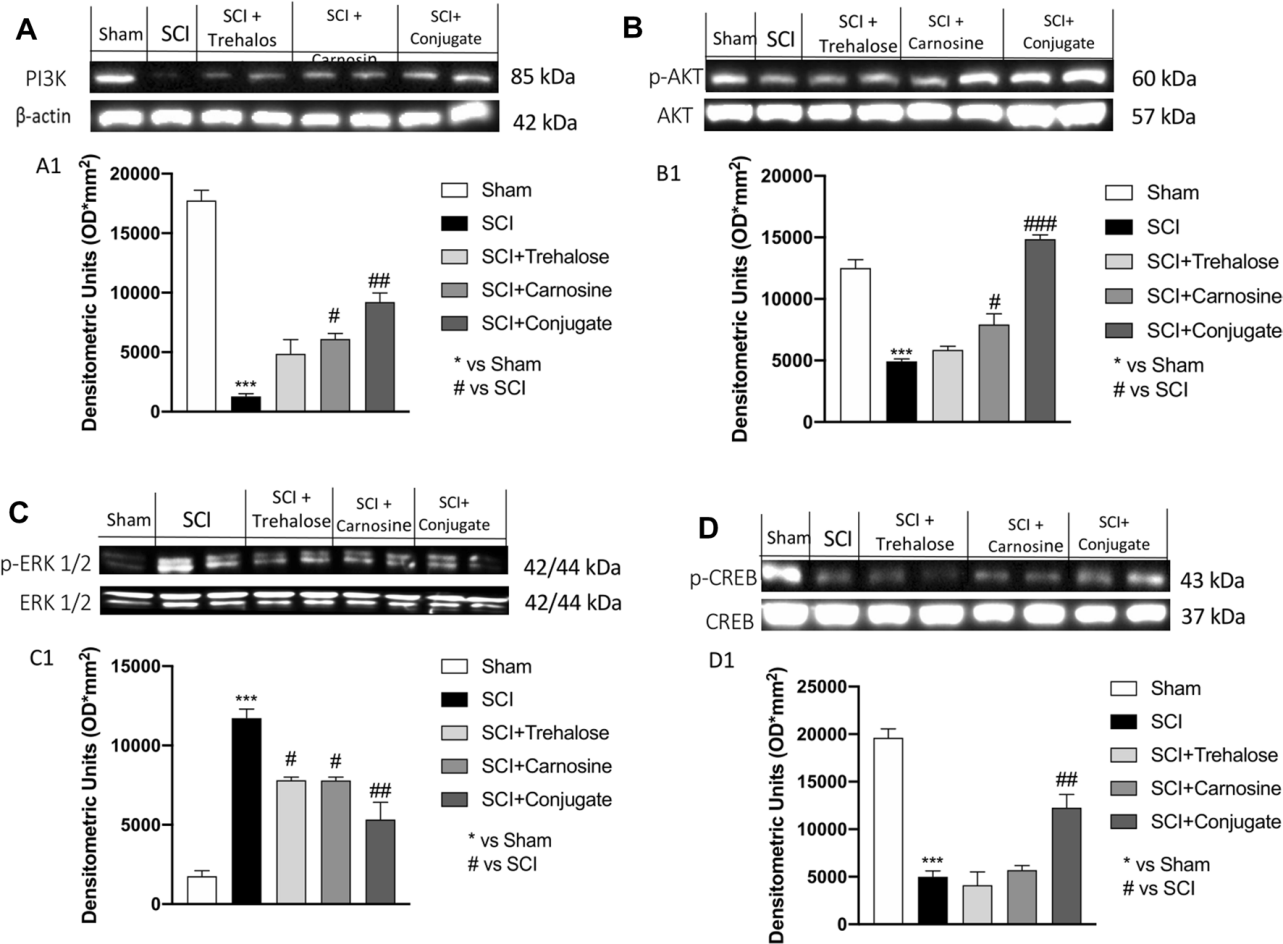
Western blot analysis. Expression level evaluation of PI3K (**A**), p-Akt (**B**), p-ERK (**C**) and p-CREB (**D**) 24 h after SCI. Densitometric analysis Panels A1, B1, C1 and D1. ***p < 0.001 versus sham; ^#^*p* < 0.05 versus SCI; ^##^*p* < 0.01 versus SCI. ^###^*p* < 0.001 versus SCI. (samples *n* = 10, three individual experiments). Data are expressed as SD

SCI is characterized by PI3K/Akt pathway downregulation (Chen et al. 2018b). To determine whether Tre–car regulates this pathway, PI3K and p-Akt/Akt protein expression levels were assayed. There was significant suppression of PI3K and p-Akt/Akt protein expression in the SCI group compared to the sham group. Although minor (in some cases not significant) changes were observed after either Tre or Car treatment alone, Tre–car significantly promoted the PI3K and p-Akt/Akt protein expression levels after SCI. These results suggest that the PI3K/Akt pathway is involved in the effects mediated by Tre and Car in SCI (Fig. 7a, b, see densitometric analyses in A1 and B1) (A1: Normal Distribution: SPSS test, Sham *W* = 0.9636, *p* = 0.826; SCI *W* = 0.9140, *p* = 0.310; SCI + Trehalose *W* = 0.8530, *p* = 0.063; SCI + Carnosine *W* = 0.8970, *p* = 0.203; SCI + Conjugate *W* = 0.9053, *p* = 0.250; *F*(4,45) = 2.59, *p* = 0.049270, one-way ANOVA method, followed by Bonferroni post hoc test for multiple comparisons) (B1: Normal Distribution: SPSS test, Sham *W* = 0.8895, *p* = 0.167; SCI *W* = 0.8993, *p* = 0.215; SCI + Trehalose *W* = 0.9573, *p* = 0.754; SCI + Carnosine *W* = 0.9134, *p* = 0.305; SCI + Conjugate *W* = 0.8577, *p* = 0.072; *F*(4,45) = 2.45, *p* = 0.054461, one-way ANOVA method, followed by Bonferroni post hoc test for multiple comparisons). Moreover, the level of p-ERK was also investigated, showing an increase 24 h after injury compared to control mice (sham group). Treatment with Tre, Car and the conjugate significantly decreased the levels of p-ERK compared to spinal cord-injured mice (Fig. 7c, see densitometric analysis in C1; Normal Distribution: SPSS test, Sham *W* = 0.8632, *p* = 0.083; SCI *W* = 0.9176, *p* = 0.338; SCI + Trehalose *W* = 0.8910, *p* = 0.174; SCI + Carnosine *W* = 0.9039, *p* = 0.242; SCI + Conjugate *W* = 0.9349, *p* = 0.497; *F*(4,45) = 2.49, *p* = 0.056585, one-way ANOVA method, followed by Bonferroni post hoc test for multiple comparisons). In addition, since the CREB factor is recognized as a positive regulator of Bcl-2 induction by negatively influencing the apoptosis (Freeland et al. 2001), we investigated its expression level after SCI and in response to our treatments. Quantitative analysis of western blots revealed that treatment with Tre–car significantly increased p-CREB expression levels compared to SCI-injured mice, whereas Tre or Car individual treatment did not affect p-CREB expression after SCI (Fig. 7d, see densitometric analysis in D1; Normal Distribution: SPSS test, Sham *W* = 0.8655, *p* = 0.089; SCI *W* = 0.9295, *p* = 0.443; SCI + Trehalose *W* = 0.9673, *p* = 0.864; SCI + Carnosine *W* = 0.9365, *p* = 0.515; SCI + Conjugate *W* = 0.9089, *p* = 0.274; *F*(4,45) = 1.27, *p* = 0.294297, one-way ANOVA method, followed by Bonferroni post hoc test for multiple comparisons).

#### Overexpression of BDNF and GDNF is Induced by Tre–Car After SCI

**Fig. 8:**
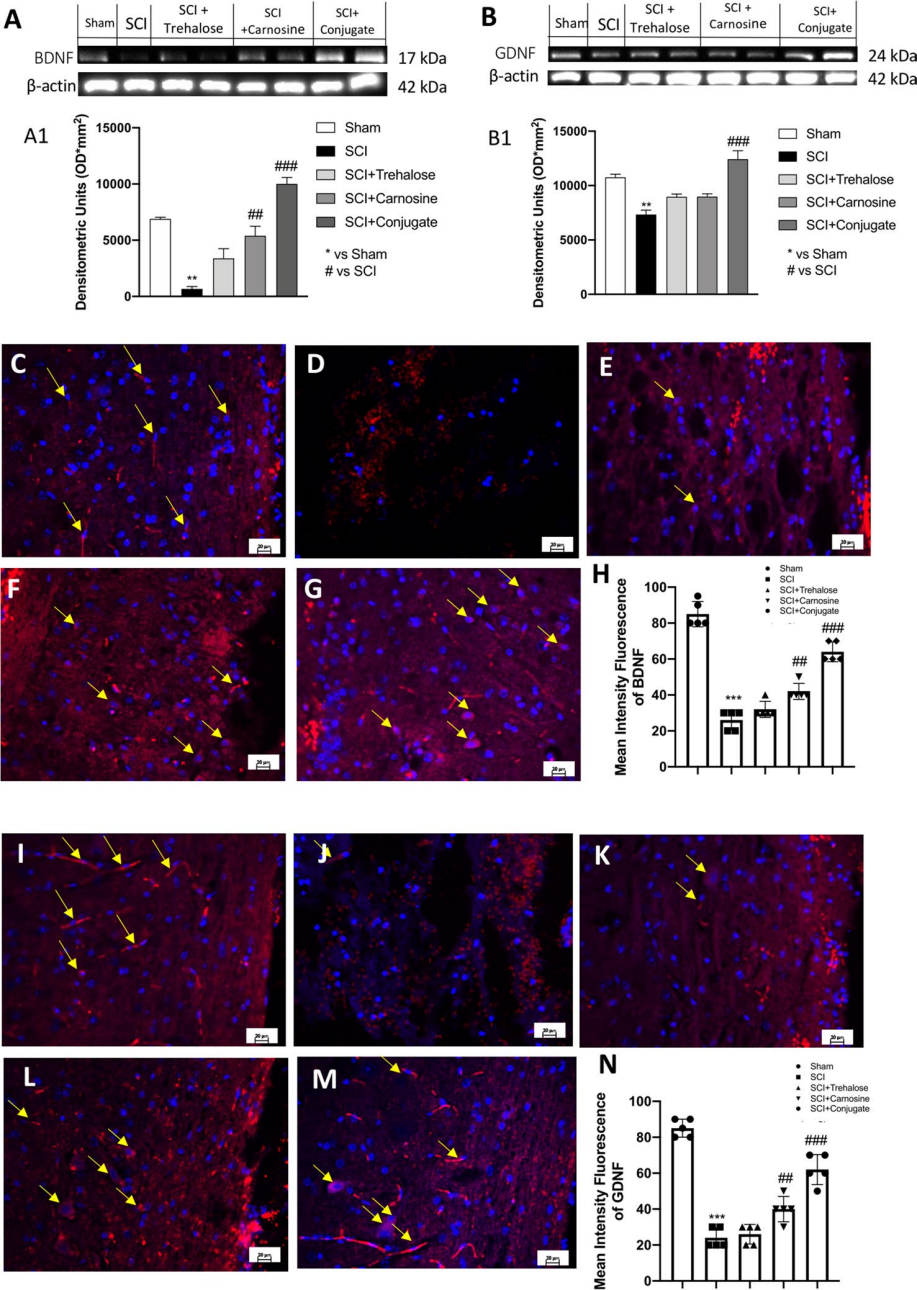
Effects of Tre–car on BDNF and GDNF. Western blot analysis showing the expression of BDNF (**A**) and GDNF (**B**) 24 h after SCI. Densitometric analysis is shown in A1 and B1. A substantial decrease in BDNF-positive staining was observed in spinal cord tissues collected from mice 24 h after SCI compared to sham mice (**C**, **D**). Treatment with Tre and Car increased the positive staining of BDNF (**E**, **F**). (samples *n* = 10, three individual experiments). Tre–car treatment further increased the positive staining of BDNF after SCI (**G**). Basal GDNF-positive staining was observed in sham mouse spinal cord tissues compared to the decreased value found in SCI mice (**I**, **J**). Treatment with Tre and Car slightly increased the positive staining of GDNF (**K**, **L**). Tre–car treatment induces a significant increase in positive staining of GDNF after SCI (**M**). Quantitative panels of BDNF and GDNF (**H**, **N**). ***p < 0.001 versus sham; ^##^*p* < 0.01 versus SCI. ^###^*p* < 0.001 versus SCI. (samples *n* = 10, three individual experiments). Data are expressed as SD

In the context of SCI, targeting neurotrophins may provide support for understanding and resolving trauma due to the neuroprotective and growth-promoting effects of these compounds (Keefe et al. 2017). Thus, expression of BDNF and GDNF proteins in the spinal cord was assayed by western blot analysis 24 h after SCI. In contrast to the Tre and Car treatment results, Tre–car induced BDNF expression levels that were significantly higher than those in the SCI group (Fig. 8a, see densitometric analysis in A1; Normal Distribution: SPSS test, Sham *W* = 0.9023, *p* = 0.232; SCI *W* = 0.8833, *p* = 0.142; SCI + Trehalose *W* = 0.9395, *p* = 0.548; SCI + Carnosine *W* = 0.8722, *p* = 0.106; SCI + Conjugate *W* = 0.8809, *p* = 0.133; *F*(4,45) = 2.78, *p* = 0.038122, one-way ANOVA method, followed by Bonferroni post hoc test for multiple comparisons). GDNF results exhibited the same trend (Fig. 8b, see densitometric analysis B1; Normal Distribution: SPSS test, Sham *W* = 0.8831, *p* = 0.142; SCI *W* = 0.8603, *p* = 0.077; SCI + Trehalose *W* = 0.9339, *p* = 0.487; SCI + Carnosine *W* = 0.8694, *p* = 0.200; SCI + Conjugate *W* = 0.9053, *p* = 0.250; *F*(4,45) = 2.43, *p* = 0.061328, one-way ANOVA method, followed by Bonferroni post hoc test for multiple comparisons). In the SCI group, BDNF expression was significantly lower than that in the sham group, and its expression was restored in the Tre–car treatment group (Fig. 8b, see densitometric analysis B1). Moreover, we assayed BDNF and GDNF immunoreactivity in spinal cord sections after SCI according to previous studies (Liang et al. 2018; Li et al. 2019b), BDNF release in the injured area of the spinal cord was absent in the SCI mouse group (Fig. 8d) compared to the sham group (Fig. 8c). An increase in the number of BDNF-positive cells was found at the site of trauma when SCI-injured mice were treated with Tre–car (Fig. 8g), highlighting the protective effects of the conjugate in comparison to its components (Fig. 8e, f) (mean of intensity fluorescence panel G; Normal Distribution: SPSS test, Sham *W* = 0.8623, *p* = 0.081; SCI *W* = 0.9065, *p* = 0.258; SCI + Trehalose *W* = 0.8946, *p* = 0.191; SCI + Carnosine *W* = 0.8946, *p* = 0.191; SCI + Conjugate *W* = 0.9197, *p* = 0.355; *F*(4,45) = 3.05, *p* = 0.026202, one-way ANOVA method, followed by Bonferroni post hoc test for multiple comparisons). Further support for the role of Tre–car in the survival of neurons was provided by GDNF immunofluorescence analysis. GDNF immunoreactivity was not found in SCI-injured mouse cells (Fig. 8j), which was different from the results in sham mice (Fig. 8i). The GDNF immunoreactivity level reached the highest value 24 h after SCI in the Tre–car-treated group (Fig. 8m) compared to the results found in both the Tre (Fig. 8k) and Car groups alone (Fig. 8l) (mean of intensity fluorescence panel N; Normal Distribution: SPSS test, Sham *W* = 0.9238, *p* = 0.390; SCI *W* = 0.9108, *p* = 0.287; SCI + Trehalose *W* = 0.8905, *p* = 0.172; SCI + Carnosine *W* = 0.9203, *p* = 0.359; SCI + Conjugate *W* = 0.8588, *p* = 0.074; *F*(4,45) = 2.45, *p* = 0.059469, one-way ANOVA method, followed by Bonferroni post hoc test for multiple comparisons). Overall, Tre–car treatment significantly affects SCI, influencing the survival of neuronal cells and contributing to their enhanced regeneration by properly modulating growth factors.

## Discussion

The findings of this study indicate that Tre–car is a multitarget molecule that protects against SCI by exerting antioxidant, anti-inflammatory, antiapoptotic and trophic activating effects in a mouse model of spinal cord injury. Conjugate protection amplifies the analogous effect of the parent moieties, including the ionophore ability due to the presence of Car, which affects zinc homeostasis by chelating the metal ion present at the micromolar level in the medium and at pathological amounts in SCI. The metal binding of Car favors the intracellular uptake of zinc, increasing the expression of ZnT1 and ZnT3, two relevant zinc transporter proteins, which belong to the SLC30A family of Zn^**2+**^ efflux transporters. In this “menage à trois” comprising the peptide, the disaccharide and the metal ion, it is possible to distinguish the different contributions responsible for the synergistic protective actions of the new molecular entity in counteracting pathological consequences of spinal cord trauma in terms of inflammatory cascade reduction at the early stage, antioxidant and antiapoptotic role and neuronal growth factor restoration. Excessive accumulation of toxic molecules produced and released by inflammatory cells may delay the recovery process. In particular, NF-kB activation is initiated by proinflammatory signals in the early phase of acute inflammation such as the interaction of TNF-α with its receptor on the cell surface (Liu et al. 2017). TNF-α-initiated signals trigger the phosphorylation of IkB-α, which leads to its detachment from NF-kB, permitting its translocation into the nucleus and driving the transcription of cytokines and proinflammatory mediators. Tre–car treatment inhibits inflammatory stress, blocking the inflammatory signaling pathways activated by NF-kB and exerting protective effects on SCI.

Redox homeostasis in the spinal cord must be maintained. The biochemical and molecular processes of secondary SCI are primarily due to oxidative and nitrosative stress that alters the abovementioned balance (Bains and Hall 2012). The primary player in oxidative stress is the superoxide radical (O_2_^⋅−^), which can react with other molecules, such as NO radicals; the reaction product of O_2_^⋅−^ with NO is peroxynitrite (ONOO^−^), which induces protein nitration by transforming tyrosine into 3-nitrotyrosine (3-Nt) (Ahsan 2013). The antioxidant activity of the dipeptide can be related to its components (L-histidine and β-alanine) that contributes to the formation of molecular adducts with different ROS in cell-free and biological assays (Pavlov et al. 1993; Bellia et al. 2011; Tamba and Torreggiani 1998; Kohen et al. 1988; Hartman et al. 1990; Prokopieva et al. 2016; Klebanov et al. 1997). l-carnosine also displays a specific and higher ability to reduce NO^.^ concentration in comparison with the constituent amino acids or their mixture in a cell-free assay; spectrophotometric and mass spectra results indicate that the direct scavenging ability against this RNS is due to carnosine/NO and carnosine/NO_2_ adduct formation (Nicoletti et al. 2007). Furthermore, Car protects astrocytes against NO-induced impairment of mitochondrial function (Nicoletti et al. 2007) and downregulates the expression of PARP-1 and PARP-2 (Spina-Purrello et al. 2010). The ability of carnosine to scavenge RNS results in its protective effects against PAR, an indicator of in vivo PARP activation, in keeping with the well-known reciprocal regulation of PARP and iNOS (Naura et al. 2009). Conversely, there are no reports on direct interactions between ROS and RNS and the nonreducing sugar trehalose. Disaccharides significantly decrease levels of iNOS in hemolysate-treated macrophage-like cells (Echigo et al. 2012), and NO that (Nazari-Robati et al. 2019) is generated immediately after SCI (Nakahara et al. 2002). In addition, trehalose counteracts the NO insult after LPS and INFγ-induced oxidative stress (Spina-Purrello et al. 2010) and downregulates PARP-1 expression in CAS-1, A-172, and SNB-19 cells (Scalia et al. 2013). The two moieties of combined Tre–car, therefore, indicate the direct (Car) and indirect (Tre) methods of protecting cells, giving rise to a synergistic effect against secondary SCI. PARP-1 is a coactivator of NF-κB and other transcription factors involved in the production of cytokines and chemokines in acute inflammation (Rothwarf and Karin 1999; Jeon et al. 2010), which represents one of the primary causes that hampers the recovery of SCI (Pineau et al. 2010). Tre inhibits inflammatory stress by suppressing NF-kB and protecting against IkB-α reduction (He et al. 2014), decreases the production of IL-1, IL-6, TNF-α, and inflammatory mediators of the acute phase of inflammation, such as NO (Taya et al. 2009). In addition to the direct interactions of Car with oxidative and nitrosative agents, different reports suggest indirect biochemical pathways used by the dipeptide to counteract both the interdependent dysregulation of redox homeostasis and inflammation and the apoptotic processes in cellular and in vivo models (Miceli et al. 2018; Cao et al. 2021; Zhang et al. 2015; Ahshin-Majd et al. 2016; Yan et al. 2019). Pretreatment with carnosine reduces the overexpression of inducible isoform nitric oxide synthase caused by nitrosative stress and modulates nitric oxide in stimulated murine RAW 264.7 macrophages (Calabrese et al. 2005; Caruso et al. 2017). In correcting the redox imbalance that characterizes early inflammation, l-carnosine enhances the nuclear transcription factor Nrf-2, which drives the antioxidant system and translocates into the nucleus. Nrf-2 activates specific genes that encode antioxidant agents that preserve redox homeostasis (Gupte et al. 2013). In stress states, NF-κB abrogates the beneficial antioxidant effect of Nrf-2 (Liu et al. 2008), through crosstalk between the two transcription factors. The ability of l-carnosine to protect against oxaliplatin-induced peripheral neuropathy has been partly attributed to the increased Nrf2 with its antioxidant machinery and the inhibition of both NF-κB and TNF-α (Yehia et al. 2019). Traumatic SCI causes the activation of NF‐κB, and the above scenario, therefore, supports the suggestion of an extension of the same biochemical pathways to the protective activity on SCI of Car and Tre–car that induces a decrease of NF-kB and an increase of IkB-α. Different studies report that l-carnosine favors cell survival by inducing the antiapoptotic marker Bcl-2 and suppressing the apoptotic marker Bax, which is associated with a decreased ratio of Bcl-2/Bax (Cheng et al. 2011; Abdel Baky et al. 2016; Wang et al. 2013). Apoptosis plays an important role in cell death in spinal cord tissue after SCI (Ding et al. 2020; Sun et al. 2020), and dipeptide findings indicate that it significantly contributes to the positive balance between the antiapoptotic marker Bcl2 and the apoptotic marker Bax shown by Tre–car in survival signaling pathway activation against SCI. The increased Nrf2 and the inhibited NF-κB (Yehia et al. 2019) may partly be related to an antiapoptotic effect, further supporting our suggestion regarding the complex signaling pathway of protection activated by l-carnosine. All assay results indicated a primary role played by Car compared to Tre in the protective effects of Tre–car on SCI; this deserves some comments primarily with respect to the effect on zinc. Large differences between Tre and Car are evident when comparing the changes in the levels of Zn transporters; SCI induces a decrease of Zn^2+^ cytosolic levels as indicated by the low value of ZnT1 and ZnT3 expression, but Car increases both ZnTs to values higher than those of the sham. Conversely, Tre did not restore control values in the case of ZnT1 and slightly increased ZnT3 expression. Zn^2+^ cannot travel across biological membranes by passive diffusion (Zhang et al. 2008); thus, the formation of the metal complex of extracellular zinc present in the SCI state with the ionophore Car induces Zn^2+^ cellular uptake, which further increases by the chelation with Tre–car, suggesting a major ionophore ability of the conjugate compared to the dipeptide. Zn^2+^ dyshomeostasis features different neuronal insults, including traumatic brain injury, stroke and seizure (Gower-Winter and Levenson 2012; Prakash et al. 2015), and a decrease in zinc serum levels is reported to be associated with trauma-induced inflammation (McClain et al. 1986), while zinc status and its time-dependent change after SCI are inadequate (Lynch et al. 2002; Farkas et al. 2019). Recently, the serum zinc levels during the acute phase of SCI were suggested to represent a predictive biomarker; namely, the metal concentration decrease was directly related to SCI severity and inversely related to the functional outcome in a mouse model (Kijima et al. 2019). Furthermore, an extension of the same approach to SCI patients shows not only that the Zn^2+^ concentration decreases in short time intervals during the initial phase after traumatic SCI but also that the metal level is correlated with the outcome and neurological impairment of injured patients, supporting zinc concentration dynamics as a predictive biomarker (Heller et al. 2020). Furthermore, a report on decreased serum Zn^2+^ levels in an SCI model with slightly increased metal concentrations within the spinal cord (Wang et al. 2011b) suggests that zinc supplementation can be an effective treatment for spinal cord ischemia/reperfusion injury in rats, highlighting (Wang et al. 2011a, 2014) the neuroprotective effect of metal ions in experimental spinal cord injury models, including functional recovery by activation of antioxidant, anti-inflammatory and anti-apoptotic processes (Li et al. 2019a, 2020; Ge et al. 2021; Lin et al. 2020). Car or Tre–car chelates extracellular Zn^2+^, and the two ionophores induce metal ion uptake, increasing the cytosolic labile metal ion pool that is involved in signaling pathways, as shown by the ZnT1 and ZnT3 expression increase. The redistribution of metal ions inside the cell activates the protein kinase cascade; phosphorylation of PI3K/Akt and CREB induces the expression of BDNF and GDNF, which contribute to secondary SCI. When we considered the PI3K/Akt/CREB/pathway, which plays a pivotal role in SCI by modulating and involving several downstream targets, we observed that treatment with the Tre–car conjugate increased the activation of PI3K and p-Akt after injury, suggesting that both pathways are involved in controlling cell survival and decreasing apoptotic cell death after trauma. Conversely, p-ERK expression, which is activated during different pathological events, including ischaemia and traumatic SCI, was decreased by Tre–car, indicating its protective effects in reducing apoptosis. Moreover, we report here that the protective abilities of l-carnosine and trehalose are mediated by PI3K/Akt-dependent CREB activation. The current findings indicate that the promotion or attenuation of these intracellular signaling cascades by Tre–car has consistent benefits for the recovery of nervous insults following spinal cord trauma. Many reports have shown that neurotrophic factors play an important role in SCI stimulating sprouting, synaptic reorganization and spinal cord regeneration (McAllister et al. 1999; Novikov et al. 1997; Ikeda et al. 2002; Namiki et al. 2000; Koda et al. 2004), while endogenous neurotrophic factor levels peak across a different range of days after spinal cord lesion during the course of the physiological response to nerve injury (Qin et al. 2006; Li et al. 2007; Yang et al. 2009). Neurotrophins exhibit a short half-life and low blood–brain barrier permeability; thus, bioengineered scaffold loaded with neurotrophins or transplantation of mesenchymal stem cell treatment has been employed to guarantee the presence of NT abundance and provide neuroprotection and some regenerative activity (Tom et al. 2018; Chung et al. 2016). In particular, BDNF, a member of the endogenous neurotrophic factor family, exerts its neuroprotective ability by binding to its specific receptor TrkB (Gupta et al. 2013). Increased production of BDNF from activated pro-inflammatory cells, including macrophages, in the injured spinal cord, has been reported to accelerate functional recovery of damaged tissue. Thus, the Tre–car conjugate prevents motor degeneration and cell death after trauma and promotes neuronal growth. Moreover, l-carnosine and Tre–Car treatment increased the expression of GDNF, a transforming growth factor-β family member that possesses tropic factors in supporting motor neurons (Allen et al. 2013). Following our treatments, an increase in GDNF expression and GDNF^+^ cells support the neuroprotective ability of Tre–car to attenuate motoneuron degeneration and promote axonal repair after spinal cord trauma.

## Conclusion Remarks

In this scenario, our findings show that l-carnosine and its conjugate with a non-innocent delivery system such as trehalose represent an alternative system of protection against oxidative stress, early inflammatory processes and apoptotic pathways induced by SCI. The dipeptide and Tre–car interaction with zinc not only induces the recovery of intracellular metal homeostasis but also activates the zinc-driven tyrosine kinase signalling pathways that produce BDNF and GDNF, employing endogenous zinc ions.

## Supplementary Information

Below is the link to the electronic supplementary material.

Supplementary file 1 (PDF 66 kb)

## Data Availability

Not applicable.
