# Supplementary figures and images for: Trehalose–Carnosine Prevents the Effects of Spinal Cord Injury Through Regulating Acute Inflammation and Zinc(II) Ion Homeostasis
Source: Cell Mol Neurobiol. 2022 Sep 19;43(4):1637–59. doi: 10.1007/s10571-022-01273-w (PMC10079760; doi:10.1007/s10571-022-01273-w)

## Supplementary materials

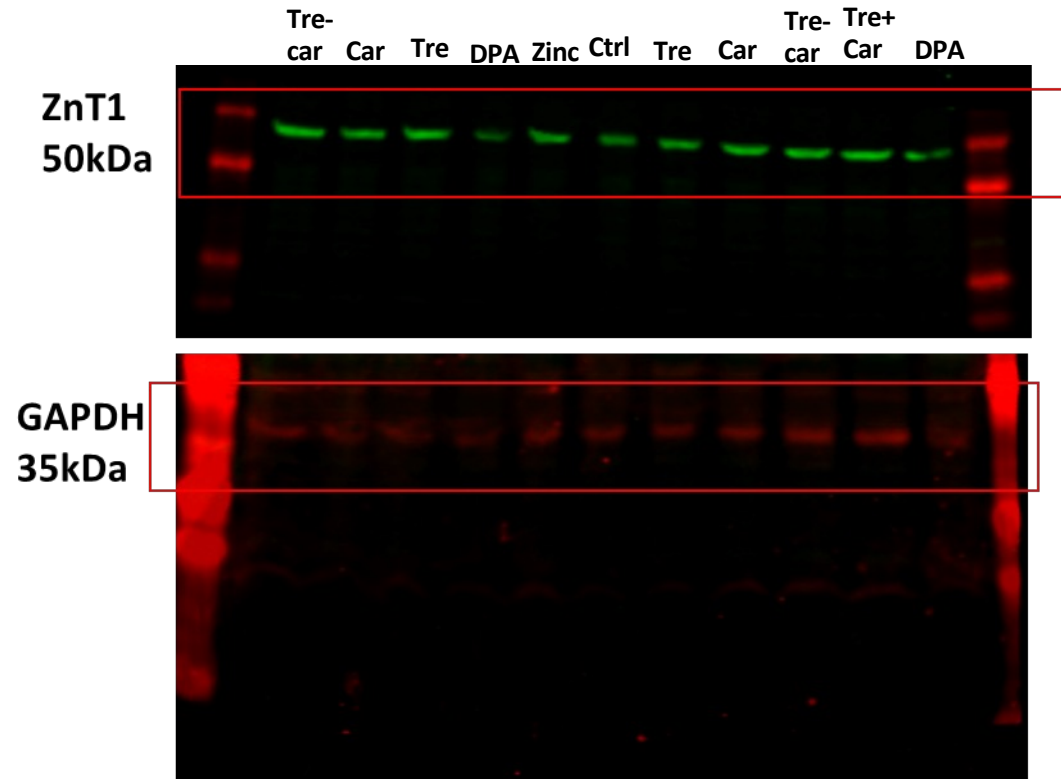

Supplement: Supplementary file 1 — (PDF 66 kb) [file 10571_2022_1273_MOESM1_ESM.pdf]
